# Floral syndromes in *Aquilegia* (Ranunculaceae) are associated with nectar- but not pollen-collecting pollinators

**DOI:** 10.1093/aob/mcaf333

**Published:** 2026-01-16

**Authors:** Anna-Sophie Hawranek, Maria von Balthazar, Marion Chartier, Jürg Schönenberger

**Affiliations:** Department of Botany and Biodiversity Research, University of Vienna, Rennweg 14, 1030 Vienna, Austria; Department of Botany and Biodiversity Research, University of Vienna, Rennweg 14, 1030 Vienna, Austria; Department of Botany and Biodiversity Research, University of Vienna, Rennweg 14, 1030 Vienna, Austria; Department of Botany and Biodiversity Research, University of Vienna, Rennweg 14, 1030 Vienna, Austria

**Keywords:** *Aquilegia*, bumble bees, columbines, floral evolution, flower morphology, pollinator functional group, machine learning, pollination syndrome, selection

## Abstract

**Background and Aims:**

Plant–pollinator interactions span a continuum from strict specialization to generalization and most flowers are visited by more than a single functional group of pollinators. However, one functional group might be more efficient than the others and thus exert stronger selective pressure on floral traits. In this study we aim to identify the evolutionary drivers of floral syndromes in the genus *Aquilegia*.

**Methods:**

We analyse floral syndromes using multivariate statistics, morphospace analyses as well as a machine learning approach (random forests), testing for the association between floral traits and documented pollinators for 28 *Aquilegia* species. In particular, we test whether pollen-collecting pollinators (small bees, large bees, syrphid flies) and nectar-collecting pollinators (large bees, hummingbirds, hawkmoths) are associated with specific floral traits. Furthermore, we test whether mixed pollination systems are reflected in floral syndrome properties.

**Key Results:**

Our results indicate that floral syndromes in *Aquilegia* are shaped mainly by nectar-collecting pollinators (and not by pollen-collecting pollinators). Flowers pollinated by large bees are mostly pendent and short-spurred; hummingbird flowers are red, with constricted spurs and short petal blades; and hawkmoth flowers are erect with long and slender spurs. Flowers pollinated by two groups of nectar-collecting pollinators show syndromes corresponding to only one of their pollinator groups.

**Conclusions:**

Despite their ubiquity, we did not find cues for selection by any of the pollen-collecting pollinators. Nevertheless, selection for traits associated with pollen-collecting pollinators, such as openly accessible stamens and a contrasted yellow floral centre (almost always present in *Aquilegia*), cannot be ruled out. Floral syndromes in *Aquilegia* are associated with nectar-collecting pollinators only, perhaps because they are more efficient at pollinating, which remains to be tested in field experiments.

## INTRODUCTION

Floral syndromes or pollination syndromes are sets of floral traits (e.g. flower shape and orientation, colour, scent, reward types) selected for by pollinating animals or abiotic agents (Delpino, 1873, 1874; Knuth and Müller, 1908; Vogel, 1954; van der Pijl, 1961; Faegri and van der Pijl, 1979; Endress, 1996; Proctor *et al*., 1996; Schemske and Bradshaw, 1999; Fenster *et al*., 2004, 2015; Ollerton *et al*., 2009; Rosas-Guerrero *et al*., 2014; Ashworth *et al*., 2015; Dellinger, 2020). In zoophilous (animal-pollinated) flowers, these traits are related to pollinator attraction (e.g. colour; Theron *et al*., 2022), pollinator constancy (e.g. rewards; Perret *et al*., 2001), and mechanical fit between flowers and pollinators (e.g. nectar tube length; Newman *et al*., 2014).

Plant–pollinator interactions typically span a continuum from strict specialization of a single pollinator species or functional group of pollinators (i.e. pollinating animals exerting similar selective pressures; Fenster *et al*., 2004; Armbruster, 2017; Smith and Kriebel, 2018; Dellinger *et al*., 2019*a*) to mixed systems. Mixed systems include bimodal pollination systems where two equally efficient pollinator functional groups exert selective pressure on a floral phenotype (Goldblatt and Manning, 2006; Dellinger *et al*., 2019*c*) and generalized systems involving several functional groups of pollinators (Rosas-Guerrero *et al*., 2014; Armbruster, 2017; Dellinger *et al*., 2019*b*). More than two-thirds of the so far investigated zoophilous angiosperms are indeed visited by several functional groups of pollinators (Ashworth *et al*., 2015; Dellinger, 2020), but only a subset of floral visitors transfer pollen efficiently (King *et al*., 2013; de Santiago-Hernández *et al*., 2019). Whether the remaining floral visitors act as occasional secondary pollinators, commensalists or even antagonists remains little studied. Further studies focusing on the interactions between flowers and their pollinators and visitors are thus crucial to understand angiosperm evolution. Pollinator shifts can lead to speciation through reproductive isolation of plant taxa (van der Niet and Johnson, 2012), and adaptation to pollinators is probably the most significant driver of flower evolution and angiosperm diversification (Harder and Johnson, 2009; van der Niet and Johnson, 2012). Early studies of pollinator shifts were conducted by Grant and Grant (1965) in Polemoniaceae and pollinator shifts are now regarded as being common (Smith, 2010; van der Niet and Johnson, 2012). Seminal examples include *Angraecum* (Orchidaceae; Wasserthal, 1997), *Disa* (Orchidaceae; Johnson *et al*., 1998), Melastomataceae (Dellinger *et al*., 2019*b*; Kopper *et al*., 2024), *Penstemon* (Plantaginaceae; Castellanos *et al*., 2003; Wilson *et al*., 2004) and *Salvia* (Lamiaceae; Claßen-Bockhoff *et al*., 2004; Wester and Claßen-Bockhoff, 2007; Kriebel *et al*., 2019, 2020).

When a plant species is pollinated by two or more functional groups of pollinators, one of these groups might be more efficient and, therefore, exert higher selective pressure on floral traits than the others (Stebbins, 1970). In this case, floral syndromes are expected to be mainly selected for by this most efficient functional group (Stebbins’ ‘most effective pollinator principle’: Stebbins, 1970; Fenster *et al*., 2004; Rosas-Guerrero *et al*., 2014). The most efficient pollinator can be termed ‘primary pollinator’, as opposed to less efficient or ‘secondary pollinators’ (Rosas-Guerrero *et al*., 2014), whereby efficiency can be tested using various methods (Reynolds and Fenster, 2008). It has been suggested that most angiosperm species with secondary pollinators exhibit floral syndromes adapted to their primary pollinators only (Rivera-Marchand and Ackerman, 2006; Martén-Rodríguez and Fenster, 2008). Note that mixed systems describe cases where a plant species is pollinated by two primary pollinators (which does not exclude the presence of secondary less efficient pollinators; Goldblatt and Manning, 2006; Dellinger *et al*., 2019*c*; Koptur *et al*., 2021; Gibson *et al*., 2022).

Secondary pollinators, even if less efficient, can also positively affect plant fitness (e.g. Wenzell *et al*., 2024) and, in some species, selection by secondary pollinators might shape floral evolution as well. Recent conceptual work on floral syndromes indeed led to the conclusion that floral phenotypes might be more accurately viewed as the sum of stronger and weaker selective pressures from all floral visitors (including also florivores and nectar robbers) and not exclusively from the primary pollinator (Ohashi *et al*., 2021). In a number of cases the secondary pollinator coincides with the probable ancestral primary pollinator (Rosas-Guerrero *et al*., 2014; Dellinger *et al*., 2019*c*). Studying the selective pressure by primary and secondary pollinators might thus greatly complement our understanding of floral evolution (e.g. Jaeger *et al*., 2023).

**Fig. 1.**
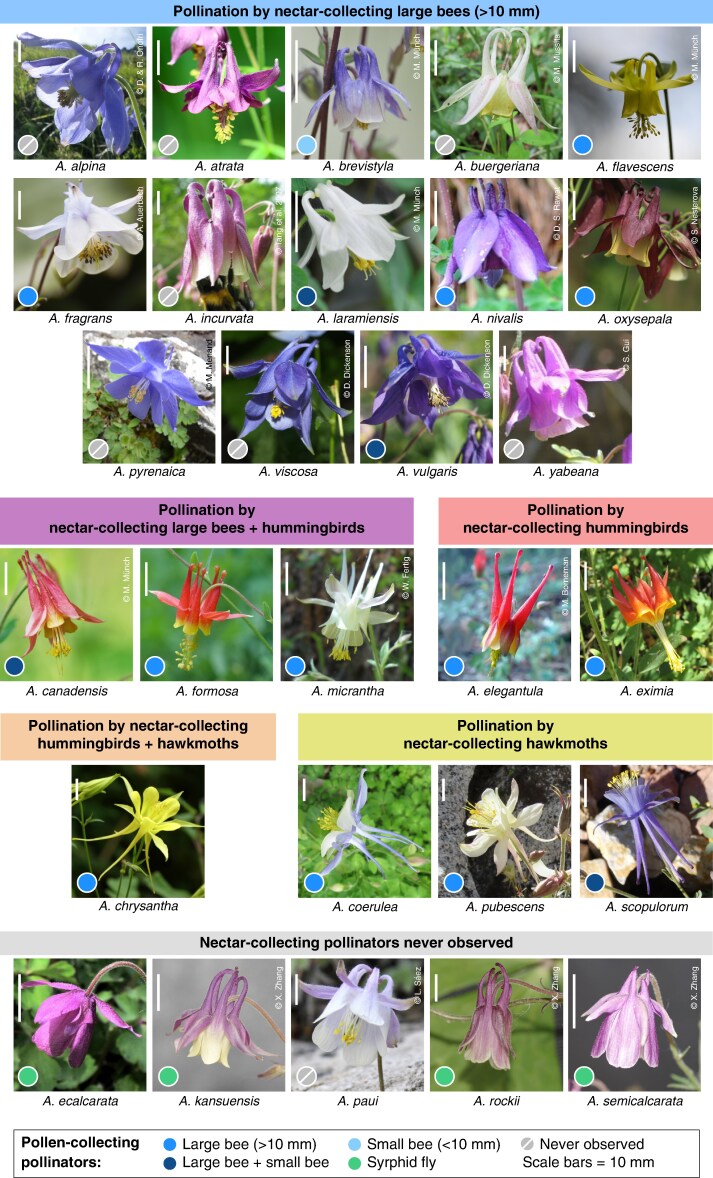
Flowers of the 28 *Aquilegia* species included in this study. Species are grouped according to their nectar-collecting pollinators. Coloured circles in the bottom left corner of each image indicate pollen-collecting pollinators. Copyright details are shown in the top right corner of each image.

Here, we study floral syndromes in relation to primary and secondary pollinators in the genus *Aquilegia* (Ranunculaceae, Ranunculales; Fig. 1). This genus comprises ca. 130 species and is distributed all over the Northern Hemisphere (Fior *et al*., 2013). *Aquilegia* flowers are polysymmetric and their perianth consists of an outer whorl of five petaloid sepals and an inner whorl of five spurred petals that extend into a laminar structure called ‘blade’ (Kramer and Hodges, 2010), ‘lamina’ (Macior, 1966) or ‘limb’ (Kramer, 2009). Each spur contains a nectariferous gland in its tip and nectar is stored in the distal part of the spurs (Hodges and Arnold, 1995; Puzey *et al*., 2012). Eight to 12 whorls of stamens and two whorls of scale-like staminodes (sterile stamens) are inserted in the floral centre surrounding a whorl of five carpels (Sharma and Kramer, 2013).

*Aquilegia* flowers are pollinated primarily by large bees (e.g. bumble bees), hummingbirds or hawkmoths, and, more rarely, by pollen-collecting syrphid flies (reviewed in von Balthazar *et al*., 2025). A first description of what later would be called floral syndromes in *Aquilegia* was given by Grant (1952; see also Grant and Grant, 1968; Grant, 1992, 1993). He divided the genus in five species complexes (*canadensis*-group, *pubescens*-group, *vulgaris*-group, *alpina*-group, *ecalcarata*-group; Grant, 1952), each defined by a particular suit of traits linked to a certain pollinator group (reviewed in Prazmo, 1965; Hodges *et al*., 2004). The evolution of floral syndromes in the North American clade (ca. 25 species) was later reconstructed on a phylogeny by Whittall and Hodges (2007), who proposed that the bee floral syndrome was ancestral in the North American clade and that from this ancestral syndrome, repeated independent morphological shifts took place toward the hummingbird syndrome and then from there toward the hawkmoth syndrome (Whittall and Hodges, 2007). Some of the floral traits in *Aquilegia*, such as flower orientation and spur length, have indeed been experimentally shown to be under selection by pollinators. For example, hawkmoths can only efficiently pollinate erect *Aquilegia* flowers with long nectar spurs (Fulton and Hodges, 1999; Hodges *et al*., 2004).

Most of the aforementioned studies on *Aquilegia* have focused on primary, nectar-collecting pollinators as selective agents. However, all studied *Aquilegia* species are pollinated or at least visited by secondary pollen-collecting insects in addition to their main pollinators. Moreover, some species are involved in mixed pollination systems including two main nectar-collecting pollinators, or one main pollen-collecting pollinator with one main nectar-collecting pollinator (reviewed in von Balthazar *et al*., 2025). For instance, *A. coerulea* is efficiently pollinated by both nectar-collecting hawkmoths and pollen-collecting bumble bees (Miller, 1981; Brunet and Holmquist, 2009) and *A. canadensis* is pollinated by both nectar-collecting bumble bees and hummingbirds (Macior, 1966).

Pollen-collecting pollinators are often assumed to be less efficient in *Aquilegia*, although there is only little information on pollinator efficiency for the genus (but see Fulton and Hodges, 1999; Brunet, 2009; Ledbetter *et al*., 2022). This lack of efficiency might occur first, because pollen-collecting pollinators might only visit flowers presenting fresh pollen and consequently not come into contact with receptive stigmas if the flowers are dichogamous (Hodges *et al*., 2004). However, dichogamy is not clearly documented in *Aquilegia* (discussed in von Balthazar *et al*., 2025), so this hypothesis might not always be supported. Second, in the case of pollen-collecting bees, part of the pollen is probably lost by grooming, which might make bees less efficient at pollen transfer than nectar-collecting animals (Thomson, 1986; Inouye *et al*., 1994; Rademaker *et al*., 1997; Thorp, 2000; Castellanos *et al*., 2003; Ashworth *et al*., 2015; Johnson and Harder, 2023). Indeed, pollen-collecting pollinators remove generally more pollen than nectar-collecting pollinators, as shown for example in *Penstemon* (Plantaginaceae; Castellanos *et al*., 2003; see also ‘Pollen Presentation Theory’ in Harder and Thomson, 1989; Thomson *et al*., 2000). In some *Aquilegia* species, however, it was found that pollen-collecting bumble bees and other bees efficiently contribute to pollination (e.g. in *A. coerulea*, otherwise pollinated by nectar-collecting hawkmoths; Brunet and Holmquist, 2009). As these secondary pollinators can be frequent in other *Aquilegia* species as well (e.g. Chartier *et al*., 2025 ), their role in shaping floral evolution and pollination syndromes should be investigated.

Since the study by Whittall and Hodges (2007) on *Aquilegia* floral syndromes, ten new studies have reported observations on pollinators for different *Aquilegia* species (see Supplementary Data Table S3, and von Balthazar *et al*., 2025). By using available pollinator observation data, it is now possible to statistically test for floral syndromes across the distribution range of the genus. Here, we combine floral morphological data and pollinator information to answer whether we can detect floral syndromes in *Aquilegia*. Since pollen- and nectar-collecting pollinators handle flowers differently (collecting pollen from anthers or probing nectar in spurs) and most of the time belong to different functional groups of pollinators (with different sizes, morphologies and behaviours), we may expect them to exert different selective pressures on floral traits. We thus analysed them separately in relation to a broad set of floral traits possibly linked to pollination, to answer the following questions: (1) Can we detect floral syndromes associated with each of the three pollen-collecting pollinator functional groups (large bees, small bees and syrphid flies)? (2) Can we detect floral syndromes associated with each of the three nectar-collecting pollinator functional groups (large bees, hummingbirds and hawkmoths)? (3) Which floral traits/trait combinations characterize these syndromes? (4) Do mixed pollination systems in *Aquilegia* exhibit mixed floral syndromes? We address these questions by applying morphospace and random forest (RF) analyses to a dataset of 15 floral traits linked to pollination for 28 *Aquilegia* species with documented pollination.

## MATERIAL AND METHODS

### Taxon sampling

We sampled all *Aquilegia* species for which we could find information about pollination (reviewed in von Balthazar *et al*., 2025). This sampling includes 28 *Aquilegia* species: ten Asian, six European and 12 North American species (Fig. 1). In addition, we distinguished two morphotypes for *A. buergeriana* as its flowers have either whitish or wine-red sepals and spurs (Toji *et al*., 2022). Similarly, both *A. flavescens* and *A. formosa* present two morphotypes each, one with spreading and one with reflexed sepals (Nold, 2003). This led to a total sampling of 28 species plus three morphotypes (Supplementary Data Table S2). To study the association of floral traits and functional groups of pollinators, we assembled two types of datasets: (1) a floral trait dataset and (2) two pollinator datasets (Table S2).

### Floral trait dataset

We selected 15 floral traits (Table 1) probably associated with plant–pollinator interactions (Supplementary Data Table S1) and including traits used to describe floral syndromes in *Aquilegia* in previous studies (e.g. Grant, 1952; Whittall and Hodges, 2007). For 21 species, trait states and values were entirely retrieved from the literature (Tables S2 and S3). For measurement, we report averages; when only ranges were available, we averaged the minimum and maximum values; and in some instances, measurements were obtained from pictures (Table S2). In addition, we used our own field observations and measurements for seven species (Tables S2 and S3). The resulting dataset contains 460 data entries, no missing data and five entries (1.08 %) of non-applicable data (spur traits for the spurless *A. ecalcarata*), treated as missing data.

**Table 1.** Floral traits linked to plant–pollinator interactions in *Aquilegia* and used to create the floral trait dataset. For more details see Supplementary Information – Section 1.1.

Trait	Trait states	State description	Example in *Aquilegia*
Floral orientation *(ordered)*	(1) Erect (2) Horizontal (3) Pendent	(1) Spur entrances directed upwards. (2) Spur entrances directed horizontally. (3) Spur entrances directed downwards.	(1) *A. coerulea* (2) *A. chrysantha* (3) *A. atrata*
Corolla colour differentiation *(unordered)*	(1) Present (2) Absent	(1) Corolla at least bicoloured. (2) Corolla unicoloured.	(1) *A. elegantula* (2) *A. atrata*
Sepal length *(metric)*		Distance from sepal bases to sepal tips (in mm).	
Sepal width *(metric)*		Length of the broadest area of the sepals (in mm).	
Sepal orientation^1^ *(ordered)*	(1) Forward (2) Spreading (3) Reflexed	(1) Sepals 0–70° relative to floral orientation. (2) Sepals 70–120° relative to floral orientation. (3) Sepals 120–180° relative to floral orientation.	(1) *A. elegantula* (2) *A. atrata* (3) *A. eximia*
Sepal colour^2^ *(unordered)*	(1) Whitish (2) Blue–purple (3) Pink–purple (4) Wine–red (5) Red (6) Yellow	According to human vision.	(1) *A. fragrans* (2) *A. vulgaris* (3) *A. rockii* (4) *A. atrata* (5) *A. elegantula* (6) *A. chrysantha*
Petal blade length *(metric)*		Distance from petal blade bases to petal blade tips (in mm).	
Petal blade colour *(unordered)*	(1) Whitish (2) Blue–purple (3) Pink–purple (4) Wine–red (5) Yellow	According to human vision.	(1) *A. fragrans* (2) *A. vulgaris* (3) *A. rockii* (4) *A. atrata* (5) *A. elegantula*
Petal spur length *(metric)*		Distance from petal spur bases to petal spur tips (in mm).	
Petal spur proportions^3^ *(unordered)*	(1) Slender (2) Stout	(1) Slender: spur lengths at least three times larger than spur widths. (2) Stout: spur lengths less than three times larger than spur widths.	(1) *A. chrysantha* (2) *A. elegantula*
Petal spur curvature^3^ *(unordered)*	(1) Absent (2) Present	(1) Spurs straight in their length. (2) Spurs curved in their length.	(1) *A. elegantula* (2) *A. chrysantha*
Petal spur constriction^3,4^ *(unordered)*	(1) Present (2) Absent	(1) Spurs constricted at around half of their length. (2) Spurs with no constriction (trumpet-shaped).	(1) *A. elegantula* (2) *A. atrata*
Petal spur hook^3^ *(unordered)*	(1) Present (2) Absent	(1) Distalmost part of the spurs hooked/curled. (2) Distalmost part of the spurs not hooked/curled.	(1) *A. atrata* (2) *A. elegantula*
Petal spur colour^2,3^ *(unordered)*	(1) Whitish (2) Blue–purple (3) Pink–purple (4) Wine–red (5) Red (6) Yellow	According to human vision.	(1) *A. fragrans* (2) *A. vulgaris* (3) *A. rockii* (4) *A. atrata* (5) *A. elegantula* (6) *A. chrysantha*
Position of reproductive organs at anthesis *(unordered)*	(1) Included (2) Exserted	(1) Anthers and stigma not protruding from the perianth. (2) Anthers and stigma protruding from the perianth.	(1) *A. vulgaris* (2) *A. atrata*

^1^Sepal orientation in *A. flavescens* and *A. formosa* ranges from spreading to reflexed (Nold, 2003), and thus we duplicated each species in the dataset and coded them once for having spreading and once for having reflexed sepals for all the analyses (two morphotypes per species).

^2^ *Aquilegia buergeriana* is polymorphic regarding sepal and spur colour (Toji *et al*., 2022). We duplicated this species in our dataset and coded it once with wine-red sepals and spurs and once with yellow sepals and spurs (two morphotypes).

^3^Since *A. ecalcarata* has no spurs (Hodges, 1997; Erst *et al*., 2017), we coded spur length with 0 mm and the other traits related to spurs as inapplicable characters.

^4^We coded the spurs of *A. incurvata* as not constricted following Tang *et al*. (2007).

### Pollinator datasets

One or several functional groups of pollinators were assigned to each *Aquilegia* species using von Balthazar *et al*. (2025) and references therein (Supplementary Data Table S3). We listed pollen-collecting pollinators and nectar-collecting pollinators separately (Table S2), because we do not expect them to exert the same types of selective pressure, and because the RF analyses do not handle two categories of class variables. Within each pollinator list, we classified pollinators based on their size and taxonomy into three functional groups. Pollen-collecting pollinators include: large bees (>10 mm in length, e.g. bumble bees and honey bees), small bees (<10 mm, e.g. sweat bees) and syrphid flies. Nectar-collecting pollinators include: large bees, hummingbirds and hawkmoths. Some (but not all) large bee species collect both pollen and nectar (von Balthazar *et al*., 2025), and in this case we included them in both analyses.

Four *Aquilegia* species (plus one morphotype) are pollinated by more than one functional group of nectar-collecting animals (Supplementary Data Table S2). Both nectar-collecting large bees and hummingbirds pollinate *A. canadensis*, *A. formosa* and *A. micrantha*, and both nectar-collecting hummingbirds and hawkmoths pollinate *A. chrysantha*. Since the pollination efficiency of these different animals is not known, these *Aquilegia* species were here considered ‘mixed’ from a pollination point of view. Note that our methods did not allow testing for mixed syndromes involving one pollen- with one nectar-collecting primary pollinator (*A. chrysantha*, *A. coerulea* and *A. eximia*) since these two types of pollinators belong to different datasets and were always analysed separately.

### Floral morphospace

To compare floral morphological differences among the 28 study *Aquilegia* species (plus three morphotypes), we analysed the morphospace formed by the floral trait dataset. From this dataset, we first computed a distance matrix. We used the mean character difference (*D*), following Sneath and Sokal (1973) and Foote (1999). *D* is a version of the Gower index, suited for datasets like ours that contain at the same time categorical ordered, categorical unordered and numerical data. The detailed calculation of *D* is given in Chartier *et al*. (2017). Calculations were made in the software R v.4.3.2 (R Core Team, 2023); functions are available upon request to M.C.

To visualize the floral morphospace, we computed a non-metric multi-dimensional scaling (nMDS; Rabinowitz, 1975) using the distance matrix with the function *metaMDS()* from the R package *vegan* (Oksanen *et al*., 2022). The goodness of fit (stress value; Clarke, 1993) was assessed with the function *stressplot()* from *vegan*. We plotted this same ordination twice: on the first plot, we mapped pollen-collecting pollinator groups, and on the second one we mapped nectar-collecting pollinator groups. This allowed us to visualize whether flowers pollinated by the same pollen- versus nectar-collecting pollinator groups cluster in the morphospace.

### Morphological differences among groups

Morphological differences among groups of *Aquilegia* species pollinated by different functional groups of pollinators were assessed in two separate tests: one with the three pollen-collecting functional groups and one with the three nectar-collecting functional groups. We performed permutational multivariate analyses of variance (PERMANOVA; Anderson, 2017), using the function *adonis2()* from the R package *vegan* (Oksanen *et al*., 2022) with 9999 permutations, and distance matrices calculated as above. Pairwise post-hoc tests were conducted using the same test with a Bonferroni correction for multiple comparisons.

We first tested for morphological differences among *Aquilegia* species grouped according to their pollen-collecting pollinators (question 1), and then according to their nectar-collecting pollinators (question 2). To deal with polymorphism in the pollinator datasets, species pollinated by two pollinator groups were duplicated and placed in each of their pollinator groups before computing a distance matrix (as explained above) and then a PERMANOVA. For the pollen-collecting pollinator analysis, we thus compared flowers pollinated by large bees, small bees and syrphid flies, and for the nectar-collecting pollinators analysis flowers pollinated by large bees, hummingbirds and hawkmoths.

### Random forest analyses

#### Predictive power of floral traits

To identify which floral traits (predictor variables) are important predictors of *Aquilegia*’s functional groups of pollinators (class variables), we conducted an RF analysis after Breiman (2001) using the R package *randomForest* (Liaw and Wiener, 2002). We analysed syndromes associated with pollen-collecting and with nectar-collecting pollinator functional groups separately (questions 1 and 2). This also allowed us to assess the predictive power of floral traits for different floral syndromes (question 3). Since polymorphism in class variables is not supported in RF, we trained our models on taxa pollinated by a single functional group only (pollen-collecting pollinators training dataset: 15 *Aquilegia* species plus two morphotypes; nectar-collecting pollinators training dataset: 19 *Aquilegia* species plus two morphotypes).

To test for the predictive power (i.e. importance) of each trait state (e.g. red, yellow and blue–purple spur colour), we converted the 11 categorical traits of the floral trait dataset into 35 dummy variables (presence/absence of each state, e.g. red spurs presence vs. absence) following Johnson (2013). In addition, we used four metric (continuous) floral traits (Table 1). We thus analysed 39 predictor variables in total.

RF analyses do not support inapplicable/missing data. We therefore removed *A. ecalcarata* from all RF analyses, since none of the predictive variables related to spur description is applicable for this spurless species.

For each analysis we computed 1000 RF (bootstrapping) of 5001 trees each. The number of predictor variables randomly sampled at each split in the generated trees (Mtry) was assessed based on the lowest out of bag (OOB) error rate (Breiman, 2002). Mtry was set to five for analysing the pollen-collecting pollinators class variables, and to six for analysing the nectar-collecting pollinators class variables. In addition to the OBB error rate, we performed sequential permutation testing using the R package *rfimptest* (Hapfelmeier and Hornung, 2023) for cross-validation.

We measured the predictive power of each predictor variable using two indices. (1) The mean decrease in accuracy assesses the loss of classification power in the RF analysis when each trait is excluded at a time (Breiman, 2001; Cutler *et al*., 2007; Martinez-Taboada and Redondo, 2020). We computed the mean decrease in accuracy for each class variable separately (e.g. large bee, hummingbird and hawkmoth) and for the entire model. (2) The mean decrease in Gini index assesses the homogeneity of categories after each split node in the classification trees (Cutler *et al*., 2007; Qi, 2012; Martinez-Taboada and Redondo, 2020).

#### Classification of species possibly involved in mixed systems

Finally, we used the trained RF models to predict classes (floral syndromes) for the four *Aquilegia* species (plus one morphotype) pollinated by two nectar-collecting functional groups (mixed pollination systems; question 4). We assessed whether these species are classified into one or more classes, i.e. if they present a floral syndrome adapted to one of their pollinators only or if they present an intermediate floral syndrome. Since we found no support for floral syndromes associated with pollen-collecting pollinators, we did not predict classes for those species pollinated by two groups of pollen-collecting pollinators.

## RESULTS

### Floral morphospace

#### Pollen-collecting pollinators

**Fig. 2.**
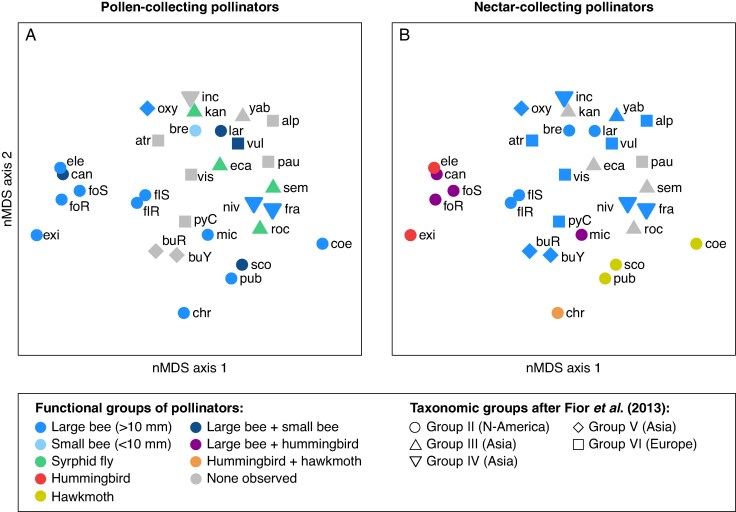
Floral morphospace ordination (nMDS) of *Aquilegia* based on 15 floral traits for 28 species (plus three morphotypes), on which are displayed, in (A), pollen-collecting pollinators, and, in (B), nectar-collecting pollinators. nMDS stress = 0.21; *R*^2^ = 0.96. See Supplementary Data Table S4 for abbreviations of species and morphotype names. See Figure S1 for the distribution of floral trait states in the morphospace.

No clear pattern emerged on the morphospace ordination when pollen-collecting pollinators were mapped (Fig. 2A; PERMANOVA: *F* = 2.257, *r*^2^ = 0.164, *P* = 0.037; all post-hoc tests were non-significant).

#### Nectar-collecting pollinators

When nectar-collecting pollinators were mapped on the floral morphospace ordination, the *Aquilegia* species pollinated by a single functional group of pollinators clearly separated into three distinct clusters (but sample size in most categories was too small to perform a PERMANOVA on these species only). Species involved in mixed-pollination systems cluster with species pollinated by a single functional group of pollinators in the space ordination (Fig. 2B). *Aquilegia canadensis* and *A. formosa* (pollinated by both large bees and hummingbirds) clustered with the bird-only-pollinated species, while *A. micrantha* (also pollinated by both large bees and hummingbirds) clustered with the large bee-only-pollinated species. On the other hand, *A. chrysantha* (pollinated by both hummingbirds and hawkmoths) clustered with the hawkmoth-only-pollinated species. When duplicating and including mixed species in the data, we detected significant differences among *Aquilegia* flowers pollinated by the three different nectar-collecting pollinator groups (PERMANOVA: *F* = 4.812, *r*^2^ = 0.256, *P* = 2 × 10^−4^). Post-hoc tests showed that large bee flowers differed significantly from hummingbird flowers (*F* = 5.741, *r*^2^ = 0.187, *P*_Bonferroni_ = 0.015), but not from hawkmoth flowers (*F* = 2.167, *r*^2^ = 0.09, *P*_Bonferroni_ = 0.198). These results also showed that hummingbird flowers did not differ significantly from hawkmoth flowers (*F* = 5.646, *r*^2^ = 0.385, *P*_Bonferroni_ = 0.097).

### Random forest analyses

#### Pollen-collecting pollinators

The RF model failed to efficiently differentiate among flowers pollinated by pollen-collecting large bees, small bees or flies (Supplementary Data Table S5), with a mean OOB error rate (±s.d.) calculated on all forests of 0.118 ± 0.006. Nevertheless, we identified some traits that tended to be associated with some pollinator groups (Fig. S2).

Flowers pollinated by syrphid flies have pink–purple spurs, sepals and petal blades and included reproductive organs (Supplementary Data Fig. S2). Large-bee-pollinated *Aquilegia* species tend to have wider sepals, while syrphid-fly-pollinated species tend to have narrower sepals.

#### Nectar-collecting pollinators

The RF model succeeded in efficiently differentiating among flowers pollinated by nectar-collecting large bees, hummingbirds and hawkmoths (Supplementary Data Table S6), with a mean OOB error rate calculated on all RFs of 0.048 ± 0.004. The classification error was zero for large bee and low (0.002) for hawkmoth flowers, but higher (0.5) for hummingbird flowers.

**Fig. 3.**
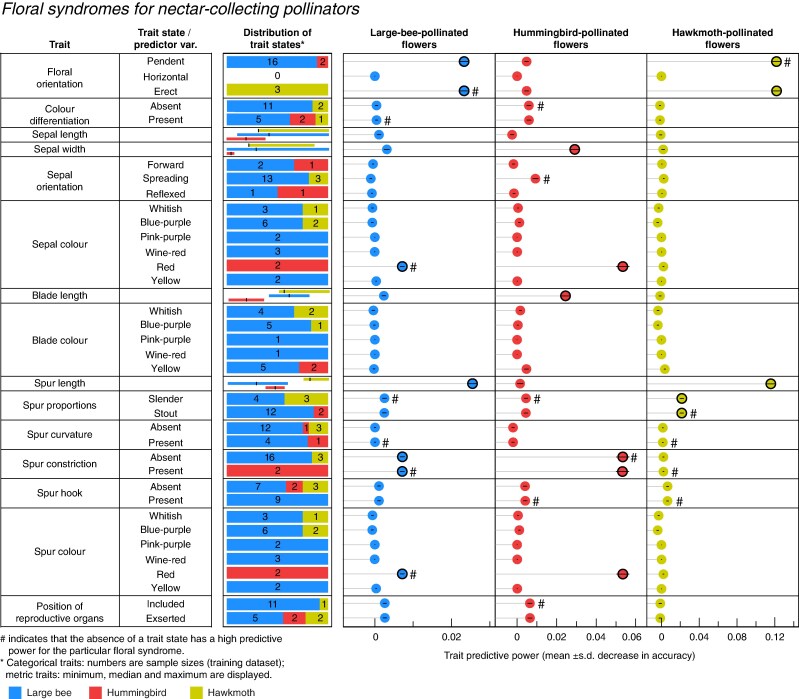
Results of the random forest (RF) analysis for nectar-collecting pollinators. Left panel: traits and RF predictor variables (trait states). Central panel: number of *Aquilegia* species (training dataset) from each pollination group displaying each trait state. For metric traits, trait distribution is given for each pollination group (minimum, median, maximum values). In blue = large bee-, in red = hummingbird-, in yellow = hawkmoth-pollinated species. Right panel: predictive power (mean decrease in accuracy across the RF) for each predictor variable. Dots circled in black correspond to predictors that we visually assessed as being most important. Note that the underlying dataset is a presence/absence matrix of trait states, and thus the absence of a trait state can also result in a high decrease in accuracy. Those cases with ‘reversed importance’ are marked with hashtags (#).

We visually identified the most important predictor variables for each pollinator group based on the mean decrease in accuracy (Supplementary Data Fig. S3) and retained five for large bee, five for hummingbird and three for hawkmoth flowers. The mean decrease in Gini index showed very similar results (Fig. S5). Large bee pollination was associated with pendent flowers, the shortest spurs (6–25 mm), no spur constriction and no red corolla (Fig. 3). Hummingbird pollination was associated with red corollas, narrow (4–5 mm) sepals, a constriction in approximately the middle of the spur length, and the shortest petal blade lengths (0–7 mm, Fig. 3). Note that spur length of species pollinated by large bees (6–25 mm) and by hummingbirds (18–24 mm) partly overlapped. Finally, hawkmoth pollination was associated with erect flowers with the longest spurs (30–38 mm), and slender spurs (Fig. 3).

In summary, the seven most important traits associated with nectar-collecting pollinators in *Aquilegia* were floral orientation, spur length, spur constriction, corolla colour, blade length, spur proportions and sepal width (Fig. 3; Table 2). The sequential permutation test supported this result (Supplementary Data Fig. S7).

**Table 2.** Predictor variables with the highest predictive power (in RF analysis), and their presence in flowers pollinated by two nectar-collecting functional groups. LB = large bee, HB = hummingbird, HW = hawkmoth. Poll. = pollinators. RF class = classification obtained in the RF analysis.

Traits (predictor variables)	Most important predictors in the RF analysis			Trait state for species in the classification analysis			
	LB flowers	HB flowers	HW flowers	*A. canadensis* Poll.: LB, HB RF class: HB	*A. chrysantha* Poll.: HB, HW RF class: LB	*A. formosa* Poll.: LB, HB RF class: HB	*A. micrantha* Poll.: LB, HB RF class LB
Short spurs	x						
Pendent flowers	x			x		x	x
Sepals not red	x				x		x
Spurs not red	x				x		x
No spur constriction	x				x		x
Red sepals		x		x		x	
Red spurs		x		x		x	
Spur constriction		x		x		x	
Narrow sepals		x					
Short blades		x		x		x	
Erect flowers			x				
Long spurs			x		x		
Slender spurs			x		x		x
Horizontal flowers					x		

#### Mixed systems for nectar-collecting pollinators

The four *Aquilegia* species pollinated by two groups of nectar-collecting animals each were classified by 98.6–100 % of the RFs in one pollinator floral syndrome only (Supplementary Data Table S7). *Aquilegia canadensis* and both morphotypes of *A. formosa*, all three pollinated by both nectar-collecting large bees and hummingbirds, were classified in the hummingbird floral syndrome in 100 % of RFs. *Aquilegia micrantha*, pollinated by both large bees and hummingbirds, was, on the contrary, classified in the large bee floral syndrome in 100 % of RFs. Finally, *A. chrysantha*, pollinated by both hummingbirds and hawkmoths, was classified in the large bee floral syndrome in 98.6 % of RFs and in the hawkmoth floral syndrome in 1.4 % of RFs. For these four species, the trait values for the predictor variables with the highest predictive power are shown in Table 2.

## DISCUSSION

We show that despite their ubiquity (von Balthazar *et al*., 2025), pollen-collecting pollinators do not seem to select for any of the investigated floral traits in *Aquilegia*. However, our analyses clearly support the existence of distinct floral syndromes associated with nectar-collecting large bees, hummingbirds and hawkmoths, respectively.

### Pollen-collecting pollinators and floral traits

Since pollen-collecting insects visit most *Aquilegia* species and are thought to pollinate them at least to some extent (e.g. Macior, 1966; Miller, 1978; Brunet *et al*., 2015; Chartier *et al*., 2025; reviewed in von Balthazar *et al*., 2025), we hypothesized that pollen-collecting large bees, small bees and syrphid flies might exert selective pressure on floral phenotypes. Also, in other lineages with flowers that offer both nectar and pollen, there are examples for which it was shown that not only nectar-collecting but also pollen-collecting pollinators significantly influence fitness (e.g. *Abronia fragrans*, Nyctaginaceae: Jaeger *et al*., 2023; *Castilleja sessiliflora*, Orobanchaceae: Wenzell *et al*., 2024). However, in *Aquilegia* we could not identify any floral syndromes statistically associated with either of the pollen-collecting pollinator groups based on the 15 floral traits included here (Table 1). A possible explanation for this finding is that pollen-collecting large bees, small bees and syrphid flies might all select for similar floral traits as all of them show a similar foraging behaviour (Inouye *et al*., 2015; our personal observations). In the case of *Aquilegia*, such traits might include exerted or at least well-accessible anthers, a yellow floral centre (petal blades, anthers) making pollen easily visible and accessible, as well as stigmas placed in proximity to the anthers, favouring contact between pollen-foraging animals and the stigmas. These characteristics are present in all *Aquilegia* species we considered here.

The only trait showing small but significant differences among the pollen-collecting groups is flower colour; more precisely, pink–purple flowers are associated with the only four syrphid fly-pollinated *Aquilegia* species in our dataset (Tang *et al*., 2007; Han *et al*., 2022). These four species belong to the same clade endemic to Asia (the *A. ecalcarata* clade or Group III *sensu*  Fior *et al*., 2013), which is the only clade for which syrphid-fly pollination has been reported so far, making it difficult to identify whether the association between fly pollination and the pink–purple corollas results from phylogenetic or selective factors. These four species are all positioned in the top right part of the space (but they overlap with other species), and their similarity might explain why the overall PERMANOVA test was significant for pollen-collecting pollinators despite the non-significance of post-hoc tests.

Another explanation for the lack of a significant association between pollen-collecting pollinators and floral traits might simply be that these pollinators are not efficient enough to select for floral traits. Pollen-collecting pollinators might even reduce male fitness by removing pollen for feeding, and their role as mutualists (secondary – less/not efficient – pollinators) or antagonists (pollen thieves) still has to be resolved (Thomson, 2003). The performance of pollen-collecting animals in *Aquilegia* is a topic of debate (Hodges *et al*., 2004; Brunet and Holmquist, 2009; Chartier *et al*., 2025) that can only be closed by measuring pollinator performance. Such measurements are unfortunately largely missing so far (but see Fulton and Hodges, 1999; Brunet, 2009; Ledbetter *et al*., 2022), leaving the question of the relevance of pollen-collecting pollinators for *Aquilegia*’s reproductive success unelucidated. Pollinator performance was measured in other systems, such as *Silene* (Caryophyllaceae), and it was shown that large bees pollinating *Silene caroliniana* are more frequent visitors and more efficient than hawkmoths, despite the greater amount of pollen loss through bee pollination (Reynolds *et al*., 2009). In *A. coerulea*, by contrast, both pollen-collecting bumble bees and nectar-collecting hawkmoths are efficient pollinators (Miller, 1981; Brunet and Holmquist, 2009).

### Floral syndromes and nectar-collecting pollinators

Our analyses identified a significant association between floral traits and different functional groups of nectar-collecting pollinators. The most important traits for characterizing the large bee, the hummingbird and the hawkmoth floral syndromes were floral orientation, and different perianth characteristics including sepal and spur colour, spur length, spur proportions and spur constriction, as well as sepal width and petal blade length (Fig. 3; Table 2). Among these traits, some trait states were either restricted to or, by contrast, never occurred with a particular group of pollinators. For example, red corollas only occurred in association with hummingbird pollination (see discussion below), long spurs were never associated with nectar-collecting bee pollination (probably because bees cannot reach the nectar in long spurred flowers, our personal observations) and pendent flowers with short spurs were never associated with hawkmoth pollination (probably because hawkmoths cannot handle pendent flowers; Miller and Willard, 1983; Fulton and Hodges, 1999). In our sampling, 17 species are pollinated by bees, but only six by hummingbirds and four by hawkmoths. These differences in sample sizes and the low sample size in general might lower the power of the RF analysis. However, this unequal distribution reflects the naturally uneven distribution of floral syndromes in the genus: while most of the ca. 130 *Aquilegia* species display a bee floral syndrome (Nold, 2003), the genus contains, in addition to our taxon sampling, only six more species with a bird floral syndrome (*A. atwoodii*, *A. barnebyi*, *A. desertorum*, *A. fosteri*, *A. shockleyi* and *A. skinneri*; Nold, 2003; Welsh *et al*., 2003) and only one to three more species with a hawkmoth floral syndrome (*A. longissima*, *A. chaplinei* and *A. hinckleyana*, all very similar and sometimes the latter two species are considered varieties of *A. chrysantha*; Nold, 2003). These taxa could unfortunately not be added to our analysis because their pollinators have not yet been documented. Furthermore, it is important to note that hummingbird- and hawkmoth-pollination are restricted to the relatively well-studied North American clade (Whittall and Hodges, 2007; Fior *et al*., 2013), whereas in the Eurasian clades, only bees have so far been documented to collect nectar. Some additional pollination systems might exist in Eurasia, given that the floral morphology of some species deviates from the bee syndrome. Examples are *A. glandulosa* [narrow spurs and, considering its large corolla diameter of 60–90 mm, short spurs (6–12 mm in length), spread petal blades and numerous stamens with brush-like arrangement; Wu *et al*., 2024a; Kaigalov *et al*., 2021], *A. parviflora* (unusual, constricted petal blades; fig. 3I in Erst *et al*., 2017) and *A. viridiflora* (yellowish-green sepals and petals; Wu *et al*., 2024b). Gathering more information on these systems will be necessary to complement our current understanding on the evolution of floral syndromes across the genus.

#### Large bee floral syndrome

We found that in *Aquilegia*, pollination by large bees is mostly associated with the shortest spurs and a pendent floral orientation, two traits traditionally included in the large bee floral syndrome described by Grant (1952), Prazmo (1965) and Whittall and Hodges (2007). This combination of traits probably facilitates the handling of flowers for large bees. Note that the same species of large bees may not only collect nectar, but also collect pollen during the same or separate visits (von Balthazar *et al*., 2025). Large bees collecting pollen and nectar during a single visit display a pollen-foraging behaviour and a nectar-foraging behaviour similar to when they only collect either pollen or nectar (our personal observations). This is why we included large bees both in the analysis on pollen-collecting pollinators and in the analysis of nectar-collecting pollinators. Investigating possible differences in behaviour among bees depending on the type of reward they collect would be interesting.

Short spurs were the most important trait defining the large bee floral syndrome. A correlation between spur and bee length has also been found across several populations of the Japanese endemic *A. buergeriana* (Toji *et al*., 2022). In general, the correlation between spur/corolla tube lengths and pollinator probing parts is very common throughout angiosperms, probably because there is a direct link between pollinator constancy and efficiency, and the pollinators’ ability to access rewards while touching the reproductive parts (Knuth and Müller, 1908; Schemske, 1976; Teräs, 1976; Inouye, 1980; Goulson, 1999; Borrell, 2005; Naghiloo *et al*., 2021). Because pedicels are quite flexible in *Aquilegia*, the pendent floral orientation might ease flower handling by bumble bees, preventing flowers from tilting under the bee’s weight as bees land and hold on to the corolla to probe spurs (as observed in the field for *A. chrysantha*; supplementary material in Chartier *et al*., 2025).

Finally, longer petal blades were described to be part of the bumble bee floral syndrome in *Aquilegia* (Grant, 1952), as pollinating bees often hold on to the perianth (Miller and Willard, 1983). The RF analysis did not confirm this hypothesis (Fig. 3), possibly because hawkmoth-pollinated flowers also present longer petal blades (see below). Yellow and blue corollas were also thought to be associated with large bee pollination in *Aquilegia* (Grant, 1952; Prazmo, 1965; Whittall and Hodges, 2007; Thairu and Brunet, 2015) as in other lineages (in *Penstemon*, Plantaginaceae: Wilson *et al*., 2004; Katzer *et al*., 2019; in *Aconitum* and *Delphinium*, Ranunculaceae: Jabbour and Renner, 2012; in the *Gambelia–Galvezia* lineage, Plantaginaceae: Ogutcen *et al*., 2017). With our data, it is the absence of the red colour that was associated with large bee pollination, probably because the red-coloured species pollinated by nectar-collecting bees all belong to mixed pollination systems and were thus not included in the RF training dataset (see also paragraph below regarding the association of red corollas with bird pollination).

#### Hummingbird floral syndrome

We found that, in *Aquilegia*, hummingbird-pollination is significantly associated with red pendent flowers with spurs constricted in their mid-section, and short to absent petal blades, four traits traditionally included in the hummingbird floral syndrome described by, for example, Grant (1952), Prazmo (1965) and Whittall and Hodges (2007). We additionally found narrow sepals as an important trait for identifying hummingbird-pollinated flowers.

Red corollas seem to always be associated with hummingbird-pollination in *Aquilegia* (Grant, 1952; von Balthazar *et al*., 2025) as in many other angiosperms (Fenster *et al*., 2004). Whether red is necessary for bird-pollination in general is a topic of debate (Grant and Grant, 1968, p. 77f; Cronk and Ojeda, 2008). In *Aquilegia* red corollas are not necessary for hummingbird-pollination, as hummingbirds also pollinate the yellow *A. chrysantha* (Miller, 1985; Chartier *et al*., 2025) and the whitish/pinkish/yellowish (Nold, 2003, plates 34–36) *A. micrantha* (Miller and Willard, 1983). Hummingbirds are opportunistic: they can learn to associate other flower colours with rewards (Wolf and Hainsworth, 1983; Fenster *et al*., 2009*b*; Chmel *et al*., 2021). It has thus been proposed that the red colour of hummingbird flowers might lower the visibility of these flowers to less efficient bees (Proctor *et al*., 1996, p. 338; Cronk and Ojeda, 2008; e.g. Lunau *et al*., 2011; Coimbra *et al*., 2020; Rodríguez-Sambruno *et al*., 2024; but see Chittka and Waser, 1997; Shrestha *et al*., 2013). In *Aquilegia*, however, bees locate and pollinate red flowers (Grant, 1952; Chartier *et al*., 2025) either because they can perceive red colours (Chittka and Waser, 1997; see also Taylor, 1984; Narbona *et al*., 2021) or because they use the brightly yellow flower centres as visual cues (von Balthazar *et al*., 2025).

The function of constricted spurs in hummingbird-pollinated *Aquilegia* flowers remains unresolved. Such constrictions might prevent the dilute low-viscosity nectar found in bird-pollinated flowers from running out of the downward opening spurs (Endress, 1996, p. 137). It could also help prevent evaporation of the nectar (but this hypothesis was not tested). In bird-pollinated *Penstemon* (Plantaginaceae; Castellanos *et al*., 2004), a constriction of the corolla is hypothesized to improve the morphological fit of the birds’ beak while excluding bees from robbing nectar, and artificially constricting the corolla in *Penstemon gentianoides* resulted in significantly fewer visits by bees (del Carmen Salas-Arcos *et al*., 2019). However, spur constriction does not prevent bees from collecting nectar in, for example, *A. canadensis* (Macior, 1966) and possible selection on spur constriction needs to be investigated in the genus.

Finally, we found short/almost absent petal blades and narrow sepals as characteristic for the hummingbird floral syndrome. Pollinating hummingbirds hover in front of *Aquilegia* flowers without grabbing floral parts (Grant and Grant, 1968; Chartier *et al*., 2025). Reduced perianth parts might thus facilitate nectar access by not being in the way of a hummingbird’s body, as do short petal lobes in hummingbird-pollinated species of, for example, *Costus* (Costaceae; Kay and Grossenbacher, 2022), *Ipomopsis* (Polemoniaceae; Grant and Grant, 1968; Grant, 1992; Grant, 1993) or *Penstemon* (Plantaginaceae; Castellanos *et al*., 2004).

Note that another potentially interesting last trait, ‘the pedicel lever-mechanism’, could not be included in our analyses because we lack descriptions for most species. This mechanism, described on *A. eximia* and observed on *A. formosa* (Grant and Grant, 1968; LoPresti *et al*., 2020; Chartier *et al*., 2025), is made possible by the flexible pedicels of most *Aquilegia* flowers and activated when the hummingbirds insert their beak into a spur, levering the entire pendent flower into a more or less horizontal position, and allowing for precise placement of pollen on the bird’s body (LoPresti *et al*., 2020).

#### Hawkmoth floral syndrome

We found that hawkmoth-pollination is significantly associated with erect flowers with long, slender spurs, three traits traditionally included in the hawkmoth floral syndrome described by Grant (1952), Prazmo (1965) and Whittall and Hodges (2007).

Experiments indicated that hawkmoths are not capable of inserting their proboscises into pendent *Aquilegia* flowers (Grant, 1992; Hodges *et al*., 2004) and that artificially shortened spurs led to reduced pollen removal by hawkmoths because their bodies would not touch the reproductive organs (Hodges *et al*., 2004). The combination of long nectar tubes/spurs and erect flowers occurs often in hawkmoth-pollinated flowers (Baker, 1961; Johnson *et al*., 2017) such as *Angraecum sesquipedale* (Orchidaceae; Wasserthal, 1997; Arditti *et al*., 2012), *Crinum variabile* (Amaryllidaceae; Manning and Snijman, 2002), *Gladiolus longicollis* (Iridaceae; Alexandersson and Johnson, 2002), *Ipomopsis tenuituba* subsp. *latiloba* (Polemoniaceae; Grant, 1992, 1993) and *Silene caroliniana* (Caryophyllaceae; Reynolds *et al*., 2009). Abiotic factors may also have contributed to the evolution of the pendent flowers of bumble bee- and hummingbird-pollinated *Aquilegia* species: pendent flowers protect stamens and nectar from rain (Fenster *et al*., 2009*a*). The exception are hawkmoth flowers, because erect flowers are necessary for hawkmoth-pollination in *Aquilegia* (Fulton and Hodges, 1999). Contrary to Grant’s (1952) hypothesis, we did not identify petal blade length to be important for characterizing the hawkmoth syndrome, probably because this trait is also present in the large bee floral syndrome – in both cases, long petal blades might serve as a visual display for attracting pollinators.

Another important aspect of flowers adapted to hawkmoth pollination is their colour, which has to contrast with the background under low-light conditions in order to be seen by nocturnal insects (Baker, 1961; Miller *et al*., 2011). Hawkmoth-pollinated flowers are usually pale (Baker, 1961; Fenster *et al*., 2004), as also in *Aquilegia* (Miller, 1978; Grant, 1992; Thomson and Wilson, 2008). We did not find a significant association between floral colour and hawkmoth-pollination, probably because the hawkmoth-pollinated species from our training dataset all showed different colours. Furthermore, brightness might also be important (not only hue), but data are not available for most species. These aspects deserve more precise testing by measuring colour with non-human-biased methods (Lunau and Maier, 1995).

### Mixed pollination systems with nectar-collecting pollinators

Our data show that there are different ways to be mixed bee/bird-pollinated in *Aquilegia*, and that predicting pollinator types based solely on floral morphology is difficult.

The three species pollinated by both nectar-collecting large bees and hummingbirds were classified either in the hummingbird (*A. canadensis* and *A. formosa*) or in the large bee floral syndrome (*A. micrantha*). This shows that rather than evolving an intermediate phenotype, these mixed systems tend to be adapted to one (most efficient?) pollinator, as already hypothesized for *A. formosa* (Hodges *et al*., 2004; but see Chartier *et al*., 2025). Another possibility is that intermediate phenotypes would be maladaptive, potentially failing to attract any of the pollinators or to efficiently deposit pollen onto the pollinators’ bodies (Armbruster *et al*., 2004, 2009*a*, *b*; reviewed in Pélabon *et al*., 2012). Finally, it is possible that other, more finely tuned traits, not tested here, are intermediate. For example, it would be interesting to investigate whether the shape of the spur entrance of the two species *A. canadensis* and *A. formosa* allows both large bees and hummingbirds to access nectar, whereas those of *A. elegantula* and *A. eximia* (supplying nectar to hummingbirds only) prevent bees from accessing nectar (Miller and Willard, 1983). The case of *A. micrantha* is particularly interesting. Our analyses placed this species in the large-bee-pollinated group (Table 2), and it was previously proposed to have a hummingbird/hawkmoth floral syndrome (Whittall and Hodges, 2007), probably because of its pale colour and straight, medium to long spurs. However, its pendent floral orientation makes it impossible for hawkmoths to probe the spurs (Grant, 1992; Fulton and Hodges, 1999; Hodges *et al*., 2004) and this species is pollinated by large bees and hummingbirds only. In general, floral orientation is viewed as a strong constraint on pollinator positioning, as shown in *Commelina communis* (Commelinaceae; Ushimaru and Hyodo, 2005) and *Silene virginica* (Caryophyllaceae; Fenster *et al*., 2009*a*).

Finally, the RF model unexpectedly classified *A. chrysantha* in the large bee floral syndrome, although it is pollinated by both nectar-collecting hummingbirds and hawkmoths (Grant, 1952, 1976; Miller, 1985; Grant and Temeles, 1992; Chartier *et al*., 2025). *Aquilegia chrysantha* displays several similarities with other hawkmoth-pollinated *Aquilegia* species (long and slender petal spurs, no petal spur constriction and long petal blades). However, these traits overlap with those of large-bee-pollinated species together with the yellow floral colour. We assume that our RF model lacks training data to reliably identify hawkmoth flowers in *Aquilegia*, which is inevitable given the low number of hawkmoth-pollinated species in the genus. For example, adding *A. longissima* (strongly suspected to be pollinated by hawkmoths, and very similar to *A. chrysantha*; Gray, 1883; Trelease, 1883) to the training dataset leads the RF to predict *A. chrysantha* as hawkmoth-pollinated (analyses not shown because they are based on circular reasoning).

### Evolutionary significance and conclusions

Our results on floral syndromes inform us about the evolutionary history of *Aquilegia*. As shown here and as was discussed by other authors (e.g. Grant, 1952; von Balthazar *et al*., 2025), floral syndromes overlap to some extent (e.g. spur length of bee and hummingbird flowers, Fig. 3; Table 2), allowing for the evolution of mixed pollination systems and for transitions among pollinator groups. At the same time, some traits are filtering against some pollinator groups (e.g. bees cannot reach nectar in the long spurs of moth flowers; Fulton and Hodges, 1999). This probably resulted in the directional evolution of bee to hummingbird and then to hawkmoth pollination, as suggested by the ancestral state reconstruction of floral syndromes by Whittall and Hodges (2007).

Bees are believed to be the ancestral pollinators in *Aquilegia* (Whittall and Hodges, 2007; Fior *et al*., 2013) and evidence from other plant clades suggests that the ancestral pollinator (especially bees) often remains as secondary pollinators (e.g. Wilson *et al*., 2007; reviewed in Rosas-Guerrero *et al*., 2014). Bees visit (and sometimes pollinate) most of the documented *Aquilegia* species (reviewed in von Balthazar *et al*., 2025). Based on our results, we suggest that most *Aquilegia* flowers show in fact at least some traits that allow for or are adapted to bee pollination, irrespective of whether bees collect nectar and/or pollen. Unfortunately, measurements of pollinator performance (e.g. Reynolds and Fenster, 2008; Ne’eman *et al*., 2010; Park *et al*., 2016), which would allow us to distinguish between primary and secondary pollinators, are still largely lacking in *Aquilegia* (Grant, 1993; Hodges *et al*., 2004; von Balthazar *et al*., 2025; but see Fulton and Hodges, 1999; Brunet, 2009; Ledbetter *et al*., 2022). Assessing pollinator performance would also help elucidate possible selective outcomes when two distinct functional groups of pollinators collect pollen and nectar or when the same functional group of pollinators collects both pollen and nectar (which is the case for large bees in, for example, *A. canadensis* and *A. fragrans*).

Testing for the correlated evolution of floral traits and pollination systems in *Aquilegia* in a phylogenetic framework would complement our understanding of the evolution of floral morphology in the genus. Results from such analyses would, however, probably still be unrewarding due to the lack of a resolved phylogeny for the genus including all species studied for pollination so far. Nevertheless, note that, except for *A. eximia* and *A. formosa*, all North American study species pollinated by either hummingbirds or hawkmoths belong to different subclades of the North American clade (Whittall and Hodges, 2007), indicating that both hummingbird- and hawkmoth-pollination evolved more than once independently in this clade.

In the future, other floral traits could be added to floral syndrome analyses in *Aquilegia*, provided the data are available. Examples are pollen–ovule ratio (bees and flies might select for a higher pollen–ovule ratio than hummingbirds and hawkmoths; Cruden, 1977; Chouteau *et al*., 2006), corolla tissue thickness (hummingbirds might select for thicker floral tissues; Grant and Grant, 1968; while hawkmoths, with their much more slender and flexible proboscises can handle thin and fragile floral structures without damaging them; Thomson and Wilson, 2008), floral scent and nectar (understudied in the genus so far but known to be important at least for moth pollination; Heath *et al*., 1992; Knudsen and Tollsten, 1993), as well as flower shape. In particular, adding information about floral shape by applying three-dimensional (3D) geometric morphometrics (Rohlf and Marcus, 1993; Adams *et al*., 2004; Klingenberg, 2010; van der Niet *et al*., 2010; Savriama, 2018; Hsu *et al*., 2020; Quitzau *et al*., 2022), to describe the curvature of the petal blades, the 3D shape of the spur entrance or the arrangement of stamens, would help to further understand specific floral syndromes in *Aquilegia* and might provide insights into possible selective forces exerted by multiple pollinators in the case of mixed pollination systems.

## Supplementary Material

mcaf333_Supplementary_Data

## Supplementary data

Supplementary data are available at *Annals of Botany* online and consist of the following. Table S1: Floral traits associated with pollination in *Aquilegia* and used in our analyses. Table S2: Floral trait and pollinator datasets used for the analyses. Table S3: References for the floral trait and pollinator datasets. Table S4: List of included species with abbreviations and an overview of analyses. Table S5: Random forest confusion table for pollen-collecting pollinators. Table S6: Random forest confusion table for nectar-collecting pollinators. Table S7: Random forest floral syndrome predictions for mixed systems. Figure S1. Floral morphospace with each trait state shown separately. Figure S2: Results of the RF analysis for pollen-collecting pollinators. Figure S3: Mean decrease in accuracy of trait states for the large bee, hummingbird and hawkmoth floral syndromes. Figure S4: Mean decrease in accuracy and mean decrease in Gini index for each floral trait state associated with pollen-collecting pollinators. Figure S5: Mean decrease in accuracy and mean decrease in Gini index for each floral trait state associated with nectar-collecting pollinators. Figure S6: Result of the sequential permutation testing of the RFs for the pollen-collectors dataset. Figure S7: Result of the sequential permutation testing of the RFs for the nectar-collectors dataset.
