# Supplementary material for: Floral syndromes in Aquilegia (Ranunculaceae) are associated with nectar- but not pollen-collecting pollinators
Source: Ann Bot. 2026 Jan 16;137(5):1305–20. doi: 10.1093/aob/mcaf333 (PMC13197590; doi:10.1093/aob/mcaf333)
Supplement: mcaf333_Supplementary_Data [file mcaf333_supplementary_data.pdf]

SUPPLEMENTARY INFORMATION

Floral syndromes in *Aquilegia* (Ranunculaceae) are associated with nectar- but not pollen-collecting pollinators

Anna-Sophie Hawranek[1], Maria von Balthazar[1], Marion Chartier[1] and Jürg Schönenberger[1]

[1] Department of Botany and Biodiversity Research, University of Vienna, Vienna, Austria

Contents

1 Floral traits (Table S1) 2
1.1 Detailed trait description 2
Floral orientation 2
Colour differentiation 3
Sepal length 3
Sepal width 3
Sepal orientation 3
Sepal colour 4
Petal blade length 4
Petal blade colour 4
Petal spur length 4
Petal spur proportions 4
Petal spur curvature 4
Petal spur constriction 5
Petal spur hook 5
Petal spur colour 5
Position of reproductive organs at anthesis 5
1.2 Floral trait matrix, pollinators, plant species list and references (Tables S2 to S4) 6
2 Floral morphospace 10
2.1 Figure S1 10
3 Random forest 12
3.1 Figure S2, Tables S5 to S7 12
3.2 Selection of trait states with a high predictive power for nectar-collecting pollinators (Figure S3) 14
3.3 Comparison of measures for the predictive power of trait states (Figures S4 & S5) 15
3.4 Sequential permutation testing of RFs (Figures S6 & S7) 17
Literature cited 18

1 Floral traits (Table S1)

Table S1: References showing the association between *Aquilegia* flower morphology and pollinators for every trait analysed in this study.

Trait	Reference(s)
**Floral orientation**	Chase and Raven (1975), Fulton and Hodges (1999), Grant (1952), Grant and Grant (1968), Hodges et al. (2004), LoPresti et al. (2020), Prazmo (1965), Whittall and Hodges (2007)
**Corolla colour differentiation**	Grant and Grant (1968), Kramer and Hodges (2010), von Balthazar et al. (2025)
**Sepal length**	Prazmo (1965), Whittall and Hodges (2007)
**Sepal width**	Prazmo (1965)
**Sepal orientation**	Grant and Grant (1968)
**Sepal colour**	Chase and Raven (1975), Grant (1952), Grant and Grant (1968), Hodges et al. (2004), Miller and Willard (1983), Prazmo (1965), Whittall and Hodges (2007)
**Petal blade length**	Chase and Raven (1975), Grant (1952), Hodges et al. (2004), Prazmo (1965), Whittall and Hodges (2007)
**Petal blade colour**	Chase and Raven (1975), Grant (1952, 1993), Grant and Grant (1968), Hodges et al. (2004), Miller and Willard (1983), Prazmo (1965), Whittall and Hodges (2007)
**Petal spur length**	Chase and Raven (1975), Ennos (2008), Fulton and Hodges (1999), Grant (1952, 1993), Grant and Temeles (1992), Hodges et al. (2004), Miller and Willard (1983), Prazmo (1965), Whittall and Hodges (2007)
**Petal spur proportions**	Grant (1993), Grant and Grant (1968), Grant and Temeles (1992), Hodges et al. (2004), Prazmo (1965)
**Petal spur curvature**	Miller and Willard (1983), Prazmo (1965)
**Petal spur constriction**	Miller and Willard (1983)
**Petal spur hook**	Miller and Willard (1983), Grant (1952)
**Petal spur colour**	Chase and Raven (1975), Grant (1952, 1993), Grant and Grant (1968), Hodges et al. (2004), Miller and Willard (1983), Prazmo (1965), Whittall and Hodges (2007)
**Position of reproductive organs at anthesis**	Grant and Grant (1968), Prazmo (1965)

1.1 Detailed trait description

A set of 15 floral traits that are associated to plant pollinator interactions (11 categorical and four metric) were selected. Trait states were retrieved from the literature as well as from field observations and measurements. Detailed trait definitions will be given in the following.

Floral orientation

(1) erect, which means that the flower is directed upwards; (2) horizontal, the flower is in an about 90° angle relative to the pedicel; and (3) pendent, the flower is directed downwards.

Notes: Han et al. (2022) described *A. ecalcarata*, *A. kansuensis*, *A. rockii*, *A. semicalcarata* and *A. yabeana* as pendent to suberect. However, we classified these species as pendent, because the trait is continuous and the difference between pendent and suberect is not always clear.
Aquilegia nivalis can be seen as suberect as well, but we classified it as pendent like the species mentioned above.

Floral orientation was shown to be selected for by pollinators. Hummingbirds tend to prefer pendent flowers (LoPresti et al. 2020, Sapir and Dudley 2013), but have also been observed to visit differently oriented types (e.g. Araujo and Sazima (2003) in seasonally flooded plains of Southern Pantanal, Western Brazil; Rodriguez-Flores et al. (2019); reviewed in Leimberger et al. (2022)). Hawkmoths prefer erect flowers and cannot probe pendent flowers for nectar (Fulton and Hodges 1999, Grant 1992).

Colour differentiation

Binary

For this presence/absence trait we quantified whether the flower has two contrasting colours according to human vision. For example, in some species of *Aquilegia* the spurs and the blades show contrasting colours, which is reported as being adaptive to the pollinators visiting these flowers because it renders flowers more visible (Kramer and Hodges 2010).

Sepal length

Metric

The length of the sepal was considered as the distance, in mm, from the attachment point on the floral base to the tip of the sepal. Since we took this data from different literature sources, slight deviations from this general definition are expected.

Sepals in *Aquilegia* are petaloid and involved in pollinator attraction (Kramer 2009) and in addition, they provide clinging structures to some pollinators. Therefore, they are considered to play a major role in the interactions with pollinators.

Sepal width

Metric

Sepal width describes the width, in mm, of the broadest area of the sepal lamina. Slight deviations from this definition are possible due to different literature sources.

Sepal orientation

(1) forward, if the sepals are at an angle of 0-70° relative to the floral axis; (2) spreading, if the sepals are at an angle of 70-120° relative to the floral axis; and (3) reflexed, if the sepals are at an angle of 120-180° relative to the floral axis.

Sepals in a spreading position (i.e. in the same position as the petal blades) might enhance the visual cue displayed by the blades themselves. Sepals are likely reflexed to not obstruct the hovering pollinators when approaching the nectar spurs.

Sepal colour

(1) whitish; (2) blue-purple; (3) pink-purple; (4) wine-red; (5) red; and (6) yellow, according to human vision.

Traditionally, red has been associated to bird pollination, blue and yellow to bee pollination (the same is true for UV patterns, but unfortunately, there is not sufficient information available for *Aquilegia* to take it into account), whereas pale colours, such as white, which are better visible at night, have been referred to flowers that are moth pollinated (Fenster et al. 2004).

Petal blade length

Metric

The length of the blade is measured, in mm, from the spur entrance to the tip of the blade. Slight deviations are possible due to different literature sources.

Like the sepals, the petal blades are involved in pollinator attraction and in providing clinging structures while not being in the way for hovering visitors (MvB, pers. obs.).

Petal blade colour

(1) whitish; (2) blue-purple; (3) pink-purple; (4) wine-red; (5) red; and (6) yellow, according to human vision.

Petal spur length

Metric

Length of the spur, in mm, from its insertion point to the tip of the spur (in case of hooked spurs to the point that is the furthest away from the insertion point), slight deviations are possible due to various literature sources used.

Spurs in *Aquilegia* contain nectar as a reward for pollinators. Spur length is highly variable and has been shown to be selected for in plant-pollinator interactions (Whittall and Hodges 2007).

Petal spur proportions

(1) Slender, when the spur is at least three times as long as it is wide (at its widest area); and (2) stout, when the petal spur is shorter than three times petal spur width.

The length:width proportion of the spur reflects spur shape and might be selected for by pollinating animals since it allows for/restricts access to the nectar reward by different pollinators.

Petal spur curvature

Binary

Spur curvature describes whether the spurs as a whole are curved or not. Spur curvature is (1) absent, when the entire spur is straight; and (2) present, when the spur is curved almost over its entire length.

Petal spur constriction

Binary

A spur constriction is (1) present when there is a constriction in the mid-section of the spur; and (2) absent, when the spur is evenly tapering from base to tip.

Petal spur hook

Binary

A hooked spur is present if the distal-most part of the spur is strongly bent.

Petal spur colour

(1) whitish; (2) blue-purple; (3) pink-purple; (4) wine-red; (5) red; and (6) yellow, according to human vision.

Position of reproductive organs at anthesis

(1) included, when anthers and stigmas do not protrude from the perianth; and (2) exserted, when anthers and stigmas protrude from the perianth.

Depending on pollinator behaviour (hovering in hummingbirds and hawkmoths vs. landing in small and large bees), the spatial display of reproductive organs differs (Fenster et al. 2004, Fulton and Hodges 1999). We expect flowers pollinated by hovering animals to present exserted stamens, because that would allow pollen placement on the animal's body even if it does not land on the flower.

1.2 Floral trait matrix, pollinators, plant species list and references (Tables S2 to S4)

Table S2: Trait matrix of all *Aquilegia* species / morphotypes included in this study. Abbrev.: Lbee = large bee; Sbee = small bee, unkn = unknown, Bird = hummingbird, Moth = hawkmoth. Measurements in mm. *Measured from pictures.

**Species / [morphotype]**	**Abbrev.**	**Distribution**	**Pollen-collectors**	**Nectar-collectors**	**Floral orientation**	**Colour differentiation**	**Sepal length**	**Sepal width**	**Sepal orientation**	**Sepal colour**
Aquilegia buergeriana [red]	buR	Asia	unkn	Lbee	pendent	present	15-25	7-10	spreading	winered
Aquilegia buergeriana [yellow]	buY	Asia	unkn	Lbee	pendent	present	15-25	7-10	spreading	whitish
Aquilegia ecalcarata	eca	Asia	Fly	unkn	pendent	absent	12-15	4-6	spreading	pinkpurple
Aquilegia fragrans	fra	Asia	Lbee	Lbee	pendent	absent	25-30	10-22	spreading	whitish
Aquilegia incurvata	inc	Asia	unkn	Lbee	pendent	present	14-18	8	forward	pinkpurple
Aquilegia kansuensis	kan	Asia	Fly	unkn	pendent	present	19	5	spreading	pinkpurple
Aquilegia nivalis	niv	Asia	Lbee	Lbee	pendent	absent	22-30	12-15	spreading	bluepurple
Aquilegia oxysepala	oxy	Asia	Lbee	Lbee	pendent	present	20-30	8-10	forward	winered
Aquilegia rockii	roc	Asia	Fly	unkn	pendent	absent	22-32	7	spreading	pinkpurple
Aquilegia semicalcarata	sem	Asia	Fly	unkn	pendent	absent	22	9	spreading	pinkpurple
Aquilegia yabeana	yab	Asia	unkn	Lbee	pendent	absent	30	10	spreading	pinkpurple
Aquilegia alpina	alp	Europe	unkn	Lbee	pendent	absent	30-45	15-20	spreading	bluepurple
Aquilegia atrata	atr	Europe	unkn	Lbee	pendent	absent	15-25	8-9	spreading	winered
Aquilegia paui	pau	Europe	unkn	unkn	pendent	absent	7-16	3-8	spreading	whitish
Aquilegia pyrenaica cazorlensis	pyC	Europe	unkn	Lbee	pendent	absent	30	5-11	spreading	bluepurple
Aquilegia viscosa	vis	Europe	unkn	Lbee	pendent	absent	18-27	8-12	spreading	bluepurple
Aquilegia vulgaris	vul	Europe	Lbee&Sbee	Lbee	pendent	absent	18-25	10-12	spreading	bluepurple
Aquilegia brevistyla	bre	America	Sbee	Lbee	pendent	present	13-16	5-7*	spreading	bluepurple
Aquilegia coerulea	coe	America	Lbee	Moth	erect	present	26-51	8-23	spreading	bluepurple
Aquilegia canadensis	can	America	Lbee&Sbee	Bird&Lbee	pendent	present	8-18	3-8	forward	red
Aquilegia chrysantha	chr	America	Lbee	Bird&Moth	horizontal	absent	20-35	5-10	spreading	yellow
Aquilegia elegantula	ele	America	Lbee	Bird	pendent	present	7-11	3-5*	forward	red
Aquilegia eximia	exi	America	Lbee	Bird	pendent	present	20	5*	reflexed	red
Aquilegia flavescens [spreading sepals]	flS	America	Lbee	Lbee	pendent	absent	12-22	5-8	spreading	yellow
Aquilegia flavescens [reflexed sepals]	flR	America	Lbee	Lbee	pendent	absent	12-22	5-8	reflexed	yellow
Aquilegia formosa [spreading sepals]	foS	America	Lbee	Bird&Lbee	pendent	present	10-26	4-9	spreading	red
Aquilegia formosa [reflexed sepals]	foR	America	Lbee	Bird&Lbee	pendent	present	10-26	4-9	reflexed	red
Aquilegia laramiensis	lar	America	Lbee&Sbee	Lbee	pendent	absent	10-15	3-4*	spreading	whitish
Aquilegia micrantha	mic	America	Lbee	Bird&Lbee	pendent	absent	10-20	4-7*	spreading	whitish
Aquilegia pubescens	pub	America	Lbee	Moth	erect	absent	15-20	6-8*	spreading	whitish
Aquilegia scopulorum	sco	America	Lbee&Sbee	Moth	erect	absent	13-22	4-10	spreading	bluepurple

Table S2 continued.

**Species / morphotype**	**Blade length**	**Blade colour**	**Spur length**	**Spur proportions**	**Spur curvature**	**Spur constriction**	**Spur hook**	**Spur colour**	**Position of reprod. organs**
Aquilegia buergeriana [red]	10-15	yellow	14-19	slender	present	absent	absent	winered	included
Aquilegia buergeriana [yellow]	10-15	yellow	14-19	slender	present	absent	absent	whitish	included
Aquilegia ecalcarata	10-15	pinkpurple	0	NA	NA	NA	NA	NA	included
Aquilegia fragrans	15-18	whitish	15-18	slender	absent	absent	absent	whitish	included
Aquilegia incurvata	7-8	whitish	12-15	stout	absent	absent	present	pinkpurple	included
Aquilegia kansuensis	12	whitish	15	stout	absent	absent	present	pinkpurple	included
Aquilegia nivalis	10-12	bluepurple	10	slender	absent	absent	absent	bluepurple	included
Aquilegia oxysepala	10-13	yellow	15-20	stout	absent	absent	present	winered	included
Aquilegia rockii	12-16	pinkpurple	17-20	slender	present	absent	absent	pinkpurple	included
Aquilegia semicalcarata	16-26	pinkpurple	5	slender	absent	absent	absent	pinkpurple	included
Aquilegia yabeana	15	pinkpurple	25	stout	absent	absent	present	pinkpurple	included
Aquilegia alpina	14-17	bluepurple	18-25	stout	absent	absent	present	bluepurple	included
Aquilegia atrata	8-12	winered	10-15	stout	absent	absent	present	winered	exserted
Aquilegia paui	3-16	whitish	3-7	slender	absent	absent	present	whitish	included
Aquilegia pyrenaica cazorlensis	15	bluepurple	10-15	stout	present	absent	absent	bluepurple	exserted
Aquilegia viscosa	12	bluepurple	15-20	stout	present	absent	present	bluepurple	exserted
Aquilegia vulgaris	10-13	bluepurple	15-25	stout	absent	absent	present	bluepurple	included
Aquilegia brevistyla	8-10	whitish	6-10	stout	absent	absent	present	bluepurple	included
Aquilegia coerulea	15-25	whitish	30-45	slender	absent	absent	absent	bluepurple	included
Aquilegia canadensis	6-8	yellow	20-25	stout	absent	present	absent	red	exserted
Aquilegia chrysantha	8-16	yellow	40-70	slender	present	absent	absent	yellow	exserted
Aquilegia elegantula	6-8	yellow	16-20	stout	absent	present	absent	red	exserted
Aquilegia eximia	0	yellow	18-30	stout	present	present	absent	red	exserted
Aquilegia flavescens [spreading sepals]	10	yellow	6-18	stout	absent	absent	absent	yellow	exserted
Aquilegia flavescens [reflexed sepals]	10	yellow	6-18	stout	absent	absent	absent	yellow	exserted
Aquilegia formosa [spreading sepals]	3-6	yellow	10-20	stout	absent	present	absent	red	exserted
Aquilegia formosa [reflexed sepals]	3-6	yellow	10-20	stout	absent	present	absent	red	exserted
Aquilegia laramiensis	10	whitish	5-7	stout	absent	absent	present	whitish	included
Aquilegia micrantha	5-10	whitish	15-30	slender	absent	absent	absent	whitish	exserted
Aquilegia pubescens	8-12	whitish	25-40	slender	absent	absent	absent	whitish	exserted
Aquilegia scopulorum	10-12	bluepurple	25-35	slender	absent	absent	absent	bluepurple	exserted

Table S3: References per *Aquilegia* species for pollen- and nectar-collectors and floral traits analysed in this study. Further details can be found in von Balthazar et al. (2025, supplementary information).

**Species**	**Pollen-collectors**	**Nectar-collectors**	**Floral traits**
Aquilegia buergeriana	Toji et al. (2022)	Toji et al. (2022)	Erst et al. (2015), Hattori et al. (2014), Jäger et al. (2016), Nold (2003)
Aquilegia ecalcarata	Tang et al. (2007)	Han et al. (2022)	Han et al. (2022), Jäger et al. (2016), Nold (2003), Xue et al. (2019)
Aquilegia fragrans	Ara et al. (2019)	Ara et al. (2019)	Jäger et al. (2016), Mathew and Sinnott (2003), Nold (2003)
Aquilegia incurvata	Tang et al. (2007)	Tang et al. (2007)	China Botanical Gardens Joint Conservation Program (2024), Nold (2003), Tang et al. (2007)
Aquilegia kansuensis	Han et al. (2022)	-	China Botanical Gardens Joint Conservation Program (2024), Erst et al. (2017), Han et al. (2022), Xue et al. (2019)
Aquilegia nivalis	Dar et al. (2010)	-	Dar et al. (2010), Jäger et al. (2016), Nold (2003)
Aquilegia oxysepala	Misaki et al. (2018)	Misaki et al. (2018)	Erst et al. (2015), Flora of China (2024), Kurzenko (2012), Luo et al. (2018), Nold (2003)
Aquilegia rockii	Han et al. (2022)	-	Erst et al. (2017), Han et al. (2022), Nold (2003), Xue et al. (2019)
Aquilegia semicalcarata	Tang et al. (2007)	-	Erst et al. (2017), Han et al. (2022), Huang et al. (2022), Xue et al. (2019)
Aquilegia yabeana	Tang et al. (2007)	Tang et al. (2007)	Erst et al. (2017), Han et al. (2022), Nold (2003), Xue et al. (2019)
Aquilegia alpina	-	von Kirchner (1911)	Angerer and Muer (2004), Lauber et al. (2018), Nold (2003)
Aquilegia atrata	-	Müller (1881)	Jäger et al. (2016), Nold (2003), Trnkoczy (2020), MC&ASH pers. obs.
Aquilegia paui	Martinell et al. (2011b)	Martinell et al. (2011b)	Martinell (2008), Martinell et al. (2011a), Martinell et al. (2011b)
Aquilegia pyrenaica cazorlensis	-	Medrano et al. (2006)	Medrano et al. (2006), Nold (2003), Tela botanica (2011)
Aquilegia viscosa	-	Lavergne et al. (2005)	Calais (2019), Lavergne et al. (2005), Mayer (2015), Nold (2003)
Aquilegia vulgaris	Martinell et al. (2011b)	Martinell et al. (2011b)	Boufford (1993m), Jäger et al. (2016), Martinell et al. (2011b), Nold (2003), Trnkoczy (2004)
Aquilegia brevistyla	Chartier et al. (2025)	Chartier et al. (2025)	Boufford (1993a), Draper (2015), Nold (2003)
Aquilegia coerulea	Ledbetter et al. (2022)	Ledbetter et al. (2022)	Boufford (1993d), Jäger et al. (2016), Nold (2003)
Aquilegia canadensis	Macior (1966)	Macior (1966)	Boufford (1993b), Jäger et al. (2016), Nold (2003)
Aquilegia chrysantha	Chartier et al. (2025)	Chartier et al. (2025)	Boufford (1993c), Jäger et al. (2016), Miller (1985), Nold (2003), MC pers. obs.
Aquilegia elegantula	Miller (1978)	Miller (1978)	Boufford (1993e), Miller (1978), Nold (2003), Spellenberg (2020)
Aquilegia eximia	Chartier et al. (2025)	LoPresti et al. (2020)	Boufford (1993f), LoPresti et al. (2020), Nold (2003), Sikora (2015), MC pers. obs.
Aquilegia flavescens	Chartier et al. (2025)	Chartier et al. (2025)	Boufford (1993g), Jäger et al. (2016), Nold (2003), MC pers. obs.
Aquilegia formosa	Zemenick et al. (2018)	Zemenick et al. (2018)	Boufford (1993h), Jäger et al. (2016), Nold (2003)
Aquilegia laramiensis	Chartier et al. (2025)	Chartier et al. (2025)	Boufford (1993i), Nold (2003), MC pers. obs.
Aquilegia micrantha	Miller and Willard (1983)	Miller and Willard (1983)	Miller and Willard (1983), Nold (2003), Boufford (1993j)
Aquilegia pubescens	Fulton and Hodges (1999)	Fulton and Hodges (1999)	Boufford (1993k), Nold (2003), Thorsted (2001), MC pers. obs.
Aquilegia scopulorum	Chartier et al. (2025)	Chartier et al. (2025)	Boufford (1993l), Munz (1946), Nold (2003), Jäger et al. (2016), Tidestrom (1910), MC pers. obs.

Table S4: Species list and analysis overview. Pollen-coll. = pollen-collecting pollinator as a class variable. Nectar-coll. = nectar-collecting pollinator as a class variable. RF training = included in the training dataset. RF testing = classified in a category using RF (mixed systems).

**Species / morphotype**	**Author**	**Abbrev.**	**Morphospace analyses**	**RF training pollen-coll.**	**RF training nectar-coll.**	**RF testing nectar-coll.**
Aquilegia buergeriana [red]	Siebold & Zucc.	buR	x		x	
Aquilegia buergeriana [yellow]	Siebold & Zucc.	buY	x		x	
Aquilegia ecalcarata	Maxim.	eca	x			
Aquilegia fragrans	Benth.	fra	x	x	x	
Aquilegia incurvata	P.K.Hsiao	inc	x		x	
Aquilegia kansuensis	(Brühl) Erst	kan	x	x		
Aquilegia nivalis	(Falc. ex Brühl) J.R.Drumm. & Hutch.	niv	x	x	x	
Aquilegia oxysepala	Trautv. & C.A.Mey.	oxy	x	x	x	
Aquilegia rockii	Munz	roc	x	x		
Aquilegia semicalcarata	(Schipcz.) Erst	sem	x	x		
Aquilegia yabeana	Kitag.	yab	x		x	
Aquilegia alpina	L.	alp	x		x	
Aquilegia atrata	W.D.J.Koch	atr	x		x	
Aquilegia paui	Font Quer	pau	x			
Aquilegia pyrenaica cazorlensis	(Heywood) Pereda & M.Laínz	pyC	x		x	
Aquilegia viscosa	Gouan	vis	x		x	
Aquilegia vulgaris	L.	vul	x		x	
Aquilegia brevistyla	Hook.	bre	x	x	x	
Aquilegia coerulea	E.James	coe	x	x	x	
Aquilegia canadensis	L.	can	x			x
Aquilegia chrysantha	A.Gray	chr	x	x		x
Aquilegia elegantula	Greene	ele	x	x	x	
Aquilegia eximia	Van Houtte ex Planch.	exi	x	x	x	
Aquilegia flavescens [spreading sepals]	S.Watson	flS	x	x	x	
Aquilegia flavescens [reflexed sepals]	S.Watson	flR	x	x	x	
Aquilegia formosa [spreading sepals]	Fisch. ex DC.	foS	x	x		x
Aquilegia formosa [reflexed sepals]	Fisch. ex DC.	foR	x	x		x
Aquilegia laramiensis	A.Nelson	lar	x		x	
Aquilegia micrantha	Eastw.	mic	x	x		x
Aquilegia pubescens	Coville	pub	x	x	x	
Aquilegia scopulorum	Tidestr.	sco	x		x	

2 Floral morphospace

2.1 Figure S1

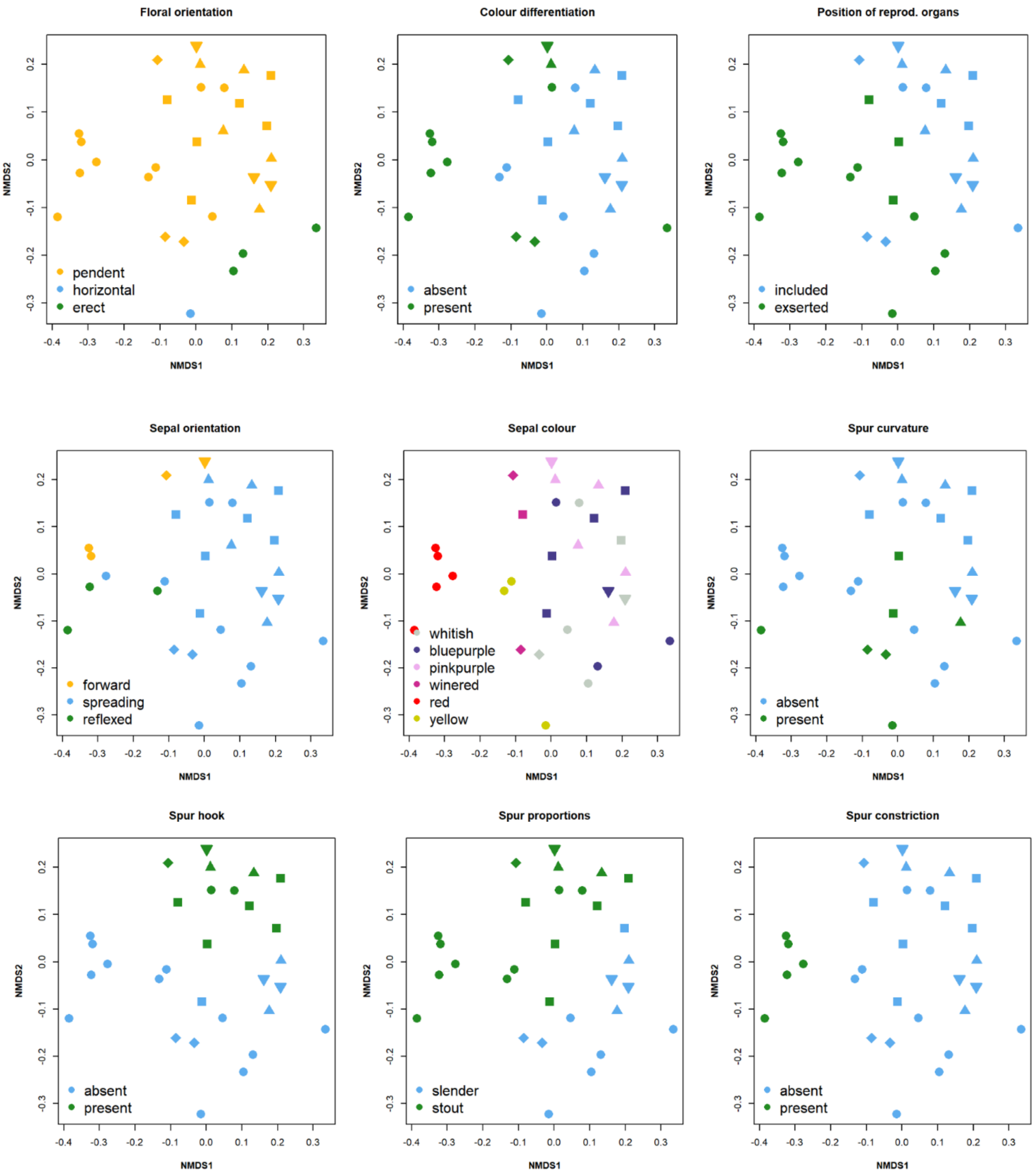

Figure S1: Floral morphospace ordination (nMDS) of *Aquilegia* based on 15 floral traits for 28 species plus 3 morphotypes, with colours representing trait states for every floral trait.

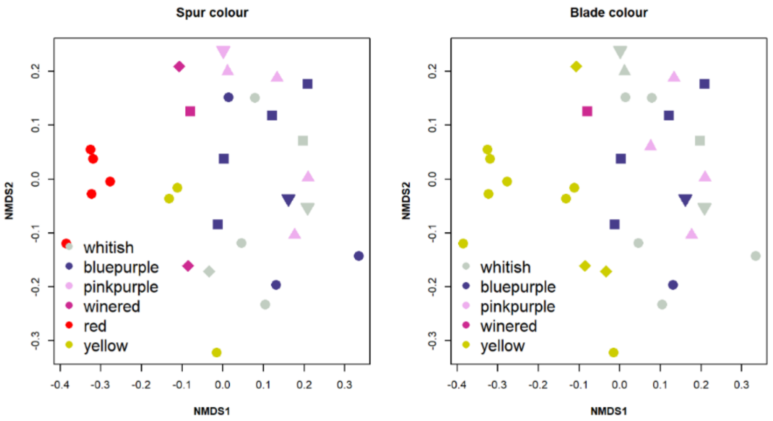

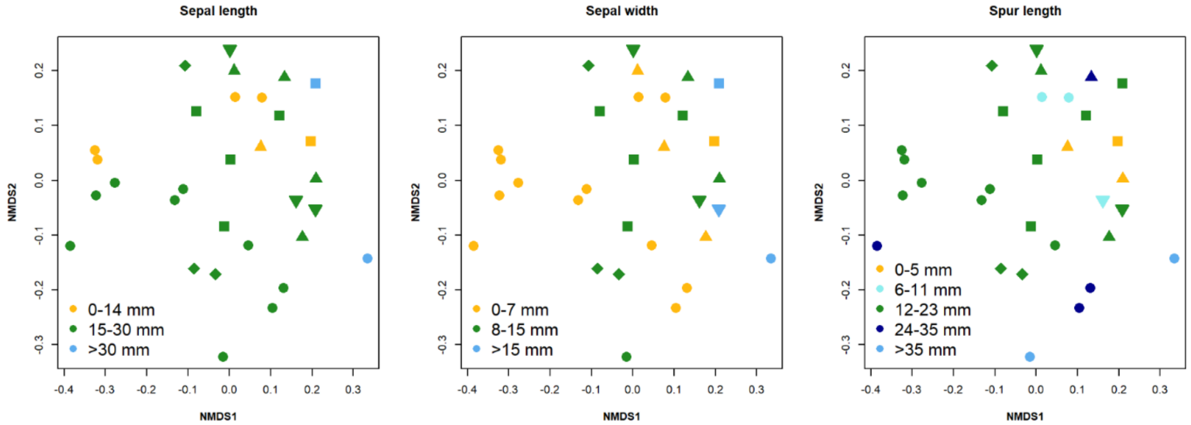

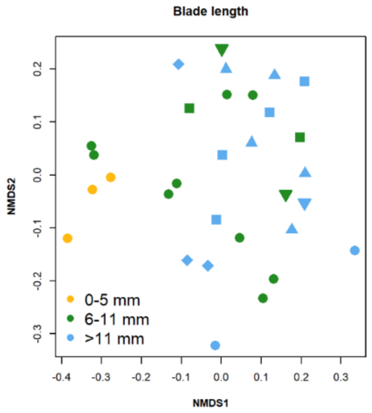

Figure S1 continued.

3 Random forest

3.1 Figure S2, Tables S5 to S7

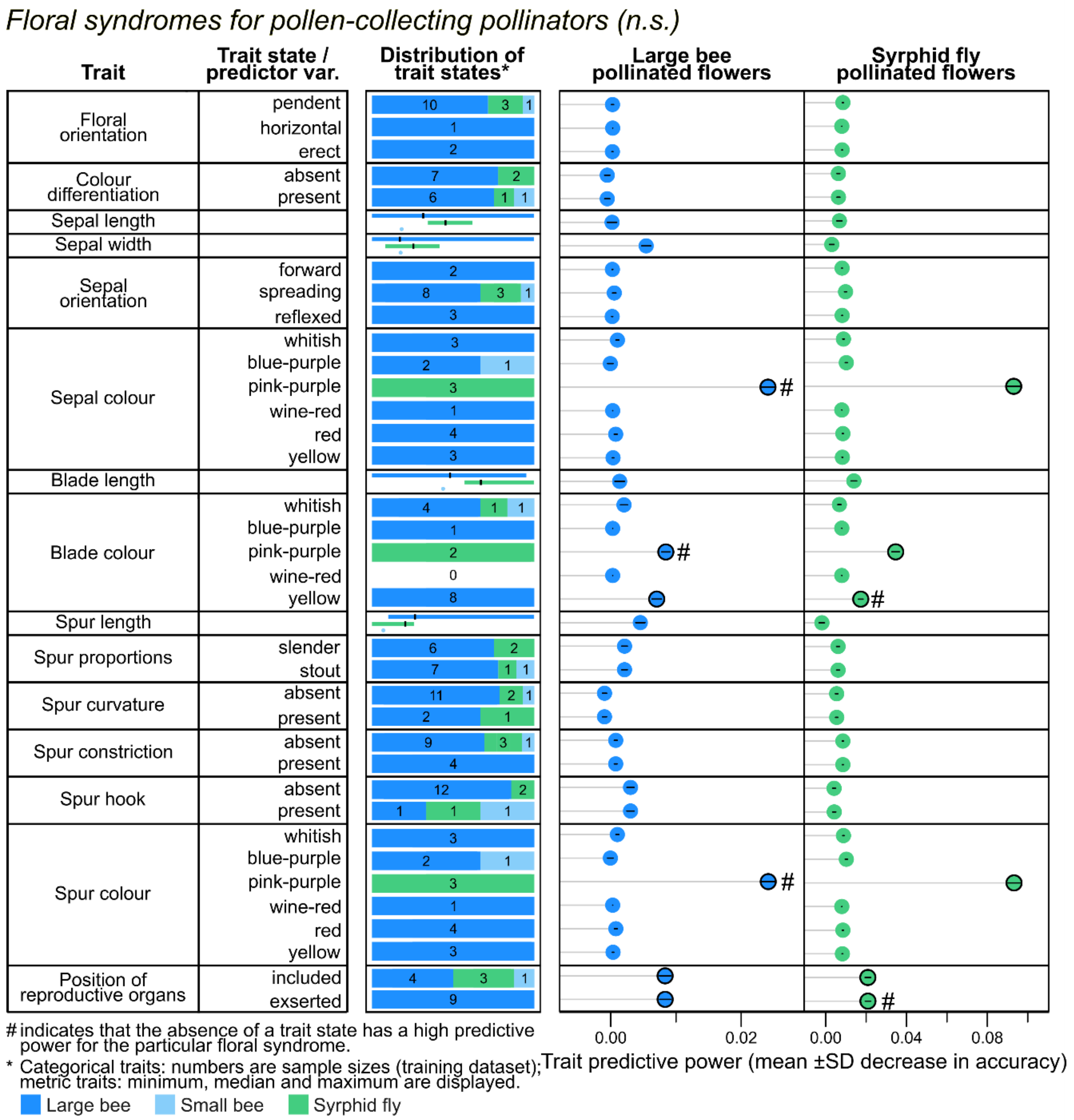

Figure S2: Results of the RF analysis for pollen-collecting pollinators. Left panel: predictor variables (trait states) and the trait category (trait) they belong to. Central panel: number of *Aquilegia* species (training dataset) from each pollination group displaying each trait state. For metric traits, distribution of these traits for each pollination group (minimum, median, maximum values). In dark blue = large bee-, in light blue = small bee-, in green = fly-pollinated species. Right panel: predictive value (mean decrease in accuracy across the RF) for each predictor variable (trait state). Dots circled in black correspond to predictors that we visually selected for as most important. Note that the underlying data is a presence/absence matrix of trait states, thus, the absence of a trait state may also result in a high decrease in accuracy and those cases with ‘reversed importance’ are marked with hashtags.

Table S5: Confusion table for pollen-collecting pollinators averaged on all forests.

	Syrphid fly	Large bee	Small bee	Classification error
**Syrphid fly**	1990	1010	0	336.6667
**Large bee**	0	13000	0	0
**Small bee**	0	1000	0	1000

Table S6: Confusion table for nectar-collecting pollinators averaged on all forests.

	Hummingbird	Large bee	Hawkmoth	Classification error
**Hummingbird**	1000	1000	0	500
**Large bee**	0	16000	0	0
**Hawkmoth**	0	6	2994	2

Table S7: Predicted nectar-collecting pollinators of mixed pollination systems calculated from out-of-bag samples of the Random Forest trained on *Aquilegia* taxa with a single type of nectar-collecting pollinators.

***Aquilegia* morphotype**	**Random forest predictions**			**Actual pollinators**
	**Hummingbird**	**Large Bee**	**Hawkmoth**	
A. canadensis	**100**	0	0	Hummingbird & large bee
A. chrysantha	0	**98.6**	1.4	Hummingbird & hawkmoth
A. formosa [reflexed sepals]	**100**	0	0	Hummingbird & large bee
A. formosa [spreading sepals]	**100**	0	0	Hummingbird & large bee
A. micrantha	0	**100**	0	Hummingbird & large bee

3.2 Selection of trait states with a high predictive power for nectar-collecting pollinators (Figure S3)

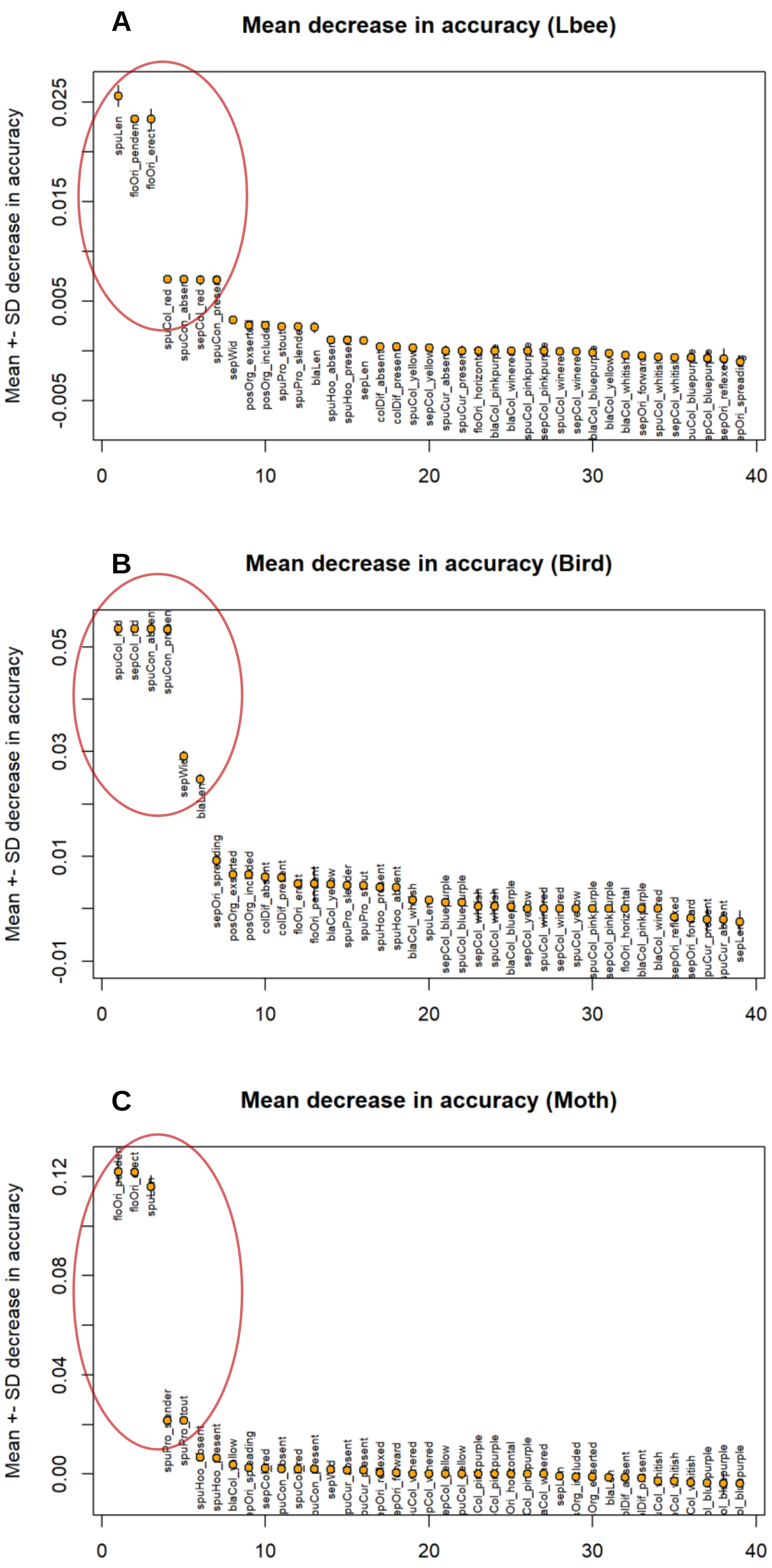

Figure S3: Selection of the traits with the highest predictive power based on the mean decrease in accuracy for every floral syndrome: (A) Large bee flowers, (B) Hummingbird flowers and (C) Hawkmoth flowers.

3.3 Comparison of measures for the predictive power of trait states (Figures S4 & S5)

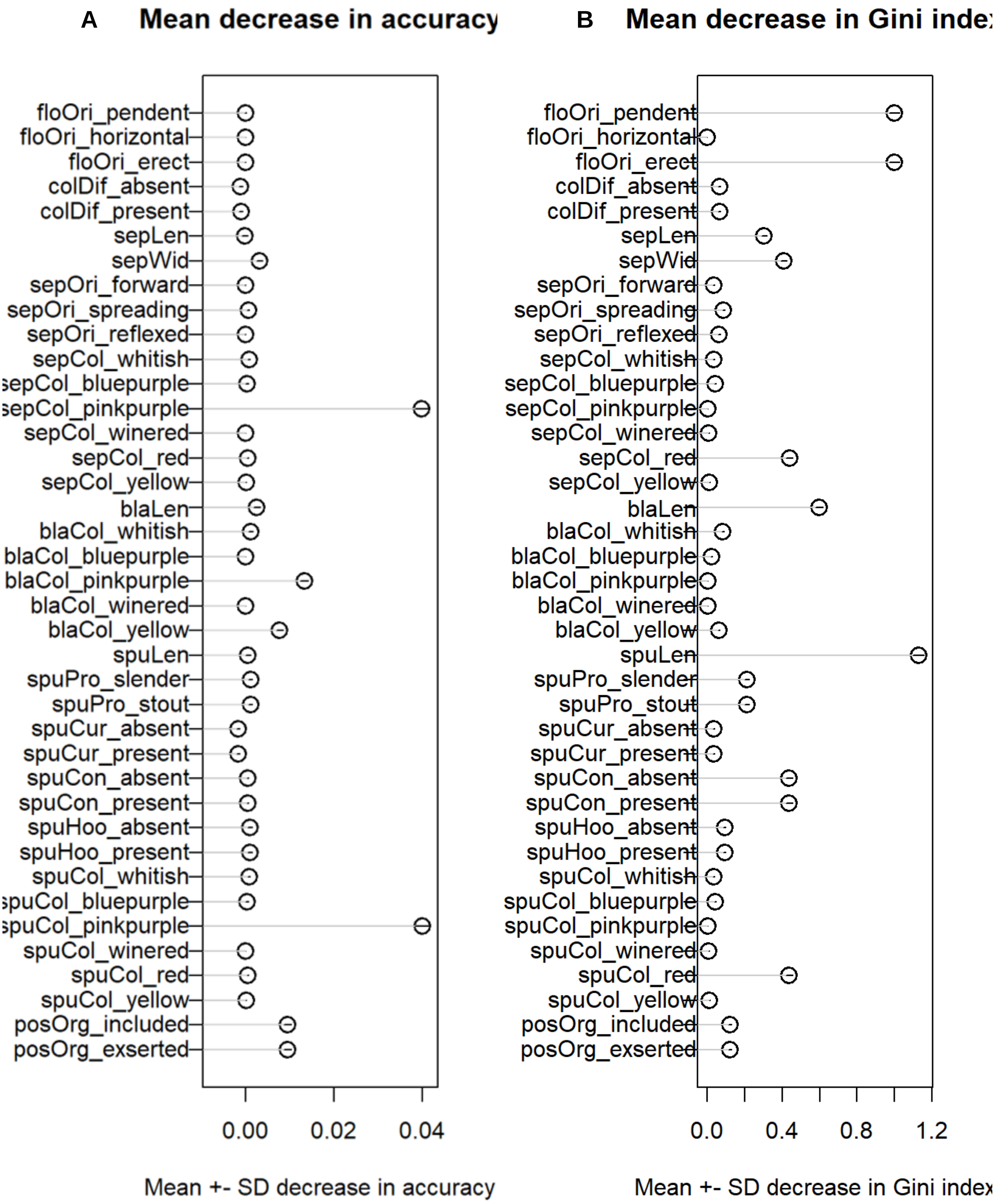

Figure S4: Predictive power of trait states for flowers pollinated by pollen-collecting pollinators of (A) mean decrease in accuracy and (B) mean decrease in Gini index.

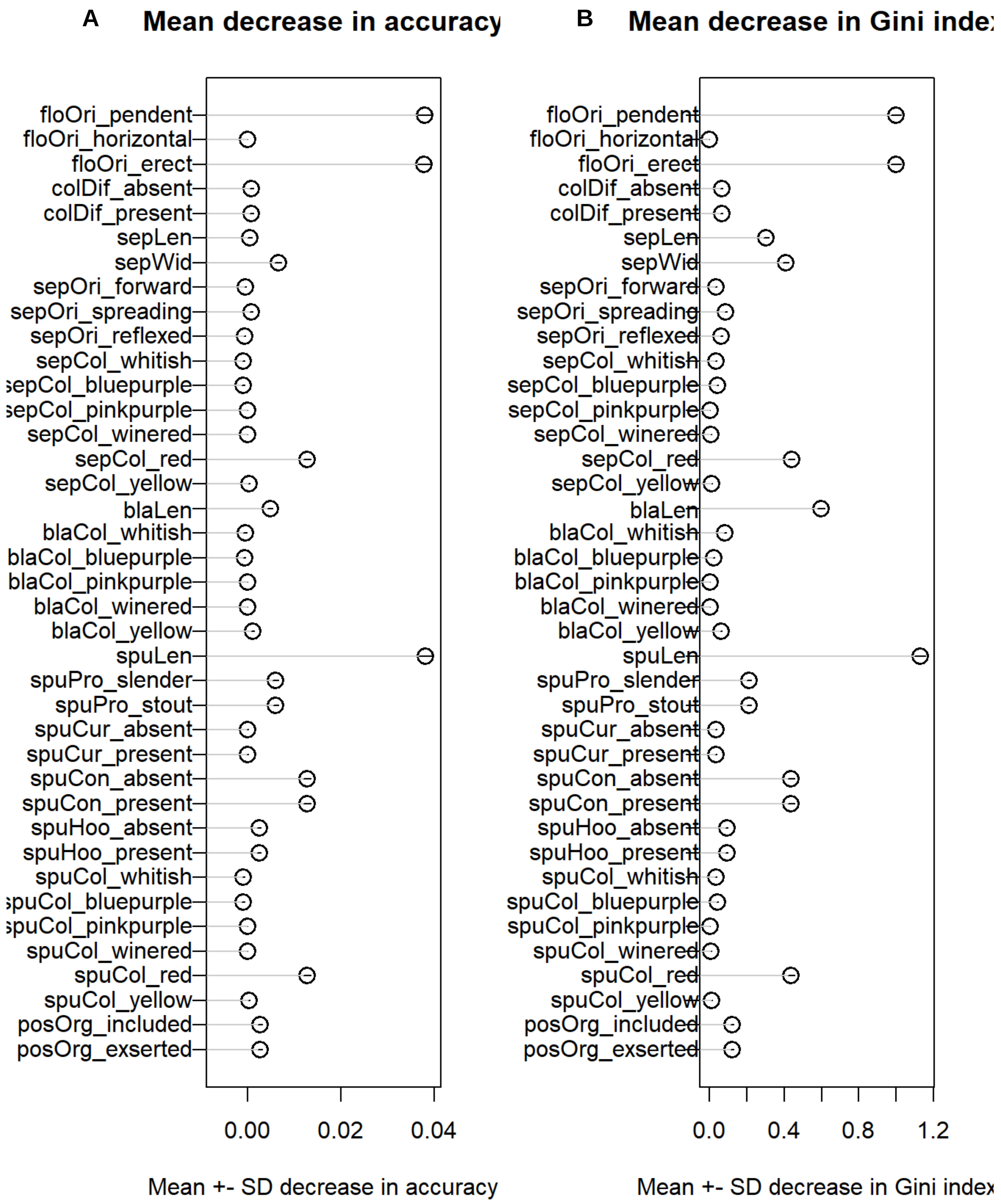

Figure S5: Predictive power of trait states for flowers pollinated by nectar-collecting pollinators of (A) mean decrease in accuracy and (B) mean decrease in Gini index.

3.4 Sequential permutation testing of RFs (Figures S6 & S7)

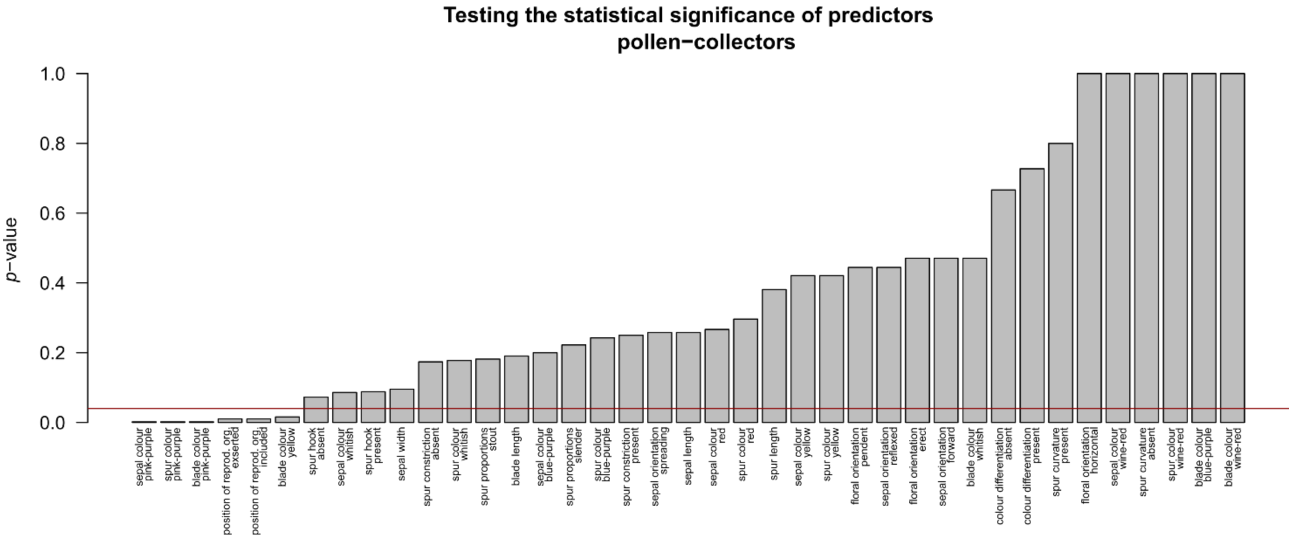

Figure S6: Result of the sequential permutation testing of the RFs for the pollen-collectors dataset. The red line denotes the default-threshold of 0.04 of the *p*-value.

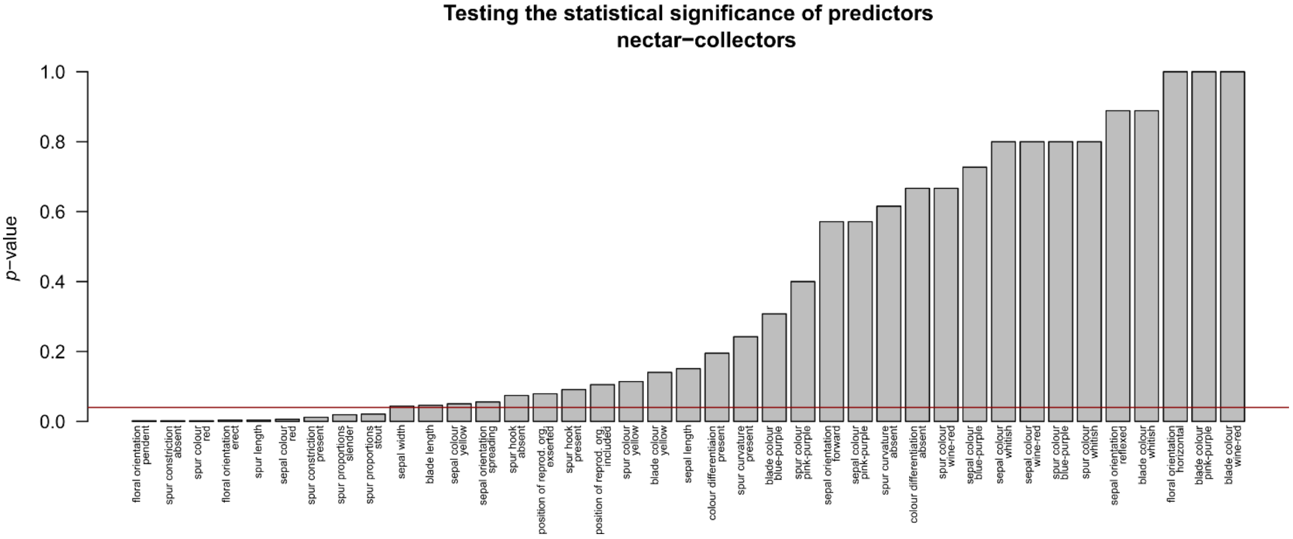

Figure S7: Result of the sequential permutation testing of the RFs for the nectar-collectors dataset. The red line denotes the default-threshold of 0.04 of the *p*-value.

Literature cited

Angerer, Oskar, and Thomas Muer. 2004. *Alpenpflanzen*. Stuttgart: Verlag Eugen Ulmer GmbH & Co.

Ara, Shoukat, Zubair Ahmad Rather, Manzoor Ahmad Paray, Rizwana Khursheed, and Munaza Yaqoob. 2019. "Bee flora of Kashmir: The Himalayan biodiversity hotspot." *Journal of Pharmacognosy and Phytochemistry* 8 (2):2172-2181.

Araujo, Andréa Cardoso, and Marlies Sazima. 2003. "The assemblage of flowers visited by hummingbirds in the "capões" of Southern Pantanal, Mato Grosso do Sul, Brazil." *Flora - Morphology, Distribution, Functional Ecology of Plants* 198 (6):427-435. doi: https://doi.org/10.1078/0367-2530-00116.

Boufford, D. E. 1993a. "*Aquilegia brevistyla* Hooker." http://floranorthamerica.org/Aquilegia_brevistyla [last accessed Dec 03, 2024].

Boufford, D. E. 1993b. "*Aquilegia canadensis* Linnaeus." http://floranorthamerica.org/Aquilegia_canadensis [last accessed Dec 03, 2024].

Boufford, D. E. 1993c. "*Aquilegia chrysantha* A.Gray." http://floranorthamerica.org/Aquilegia_chrysantha [last accessed Dec 03, 2024].

Boufford, D. E. 1993d. "*Aquilegia coerulea* E.James." http://floranorthamerica.org/Aquilegia_coerulea [last accessed Dec 03, 2024].

Boufford, D. E. 1993e. "*Aquilegia elegantula* Greene." http://floranorthamerica.org/Aquilegia_elegantula [last accessed Dec 03, 2024].

Boufford, D. E. 1993f. "*Aquilegia eximia* Van Houtte ex Planchon." http://floranorthamerica.org/Aquilegia_eximia [last accessed Dec 03, 2024].

Boufford, D. E. 1993g. "*Aquilegia flavescens* S.Watson." http://floranorthamerica.org/Aquilegia_flavescens [last accessed Dec 03, 2024].

Boufford, D. E. 1993h. "*Aquilegia formosa* Fischer ex de Candolle." http://floranorthamerica.org/Aquilegia_formosa [last accessed Dec 03, 2024].

Boufford, D. E. 1993i. "*Aquilegia laramiensis* A.Nelson." http://floranorthamerica.org/Aquilegia_laramiensis [last accessed Dec 03, 2024].

Boufford, D. E. 1993j. "*Aquilegia micrantha* Eastwood." http://floranorthamerica.org/Aquilegia_micrantha [last accessed Dec 03, 2024].

Boufford, D. E. 1993k. "*Aquilegia pubescens* Coville." http://floranorthamerica.org/Aquilegia_pubescens [last accessed Dec 03, 2024].

Boufford, D. E. 1993l. "*Aquilegia scopulorum* Tidestrom." http://floranorthamerica.org/Aquilegia_scopulorum [last accessed Dec 03, 2024].

Boufford, D. E. 1993m. "*Aquilegia vulgaris* Linnaeus." http://floranorthamerica.org/Aquilegia_vulgaris [last accessed Dec 03, 2024].

Calais, Jean-Claude. 2019. "*Aquilegia viscosa* Gouan." https://www.tela-botanica.org/bdtfx-nn-74964-illustrations [last accessed Dec 03, 2024].

Chartier, Marion, Constantin Kopper, Michael Münch, Olivia Messinger Carril, Sergio Díaz-Infante, Margarita Lachmayer, Silvia Ulrich, Joseph S. Wilson, Jürg Schönenberger, and Maria von Balthazar. 2025. "Pollination Biology and Secondary Pollinators in Seven North American *Aquilegia* Species." *International Journal of Plant Sciences* 186 (1):20-37. doi: 10.1086/733068.

Chase, Valerie C., and Peter H. Raven. 1975. "Evolutionary and ecological relationships between *Aquilegia formosa* and *A. pubescens* (Ranunculaceae), two perennial plants." *Evolution*:474-486.

China Botanical Gardens Joint Conservation Program. 2024. "Initiative for Collective Conservation in Chinese Botanical Gardens." https://image.cubg.cn/search?sort=default&text=aquilegia%20incurvata&shitu=-1&taxonId= [last accessed on Dec 03, 2024].

Dar, A. R., Reshi Zafar, G. H. Dar, and Andleeb Lubna. 2010. "Factors contributing to critically endangered status of *Aquilegia nivalis* Falc ex Jackson-an alpine endemic angiosperm in the Kashmir Himalaya, India." *International Journal of Botany* 6 (4):371-382.

Draper, Trent M. 2015. "*Aquilegia brevistyla* Photo ID: 0000 0000 0615 2774." Regents of the University of California. https://calphotos.berkeley.edu/ [last accessed Dec 03, 2024].

Ennos, R. A. 2008. "Spurred on by pollinators." *Heredity* 100:3-4.

Erst, A. S., W. Wang, S. X. Yu, K. L. Xiang, J. Wang, D. N. Shaulo, S. Smirnov, M. Kushunina, A. P. Sukhorukov, and M. Nobis. 2017. "Two new species and four new records of *Aquilegia* (Ranunculaceae) from China." *Phytotaxa* 316 (2):121-137.

Erst, Andrey S., Alexander P. Sukhorukov, Dmitriy N. Shaulo, and Alexander A. Kuznetsov. 2015. "Chorological and taxonomic notes on *Aquilegia ganboldii* Kamelin & Gubanov (Ranunculaceae) previously considered to be a Mongolian endemic." *Acta Botanica Gallica* 162 (3):165-171.

Fenster, Charles B., W. Scott Armbruster, Paul Wilson, Michele R. Dudash, and James D. Thomson. 2004. "Pollination syndromes and floral specialization." *Annual Review of Ecology, Evolution, and Systematics* 35:375-403.

Flora of China. 2024. "*Aquilegia oxysepala*. Vol. 6, p. 280." http://www.efloras.org/florataxon.aspx?flora_id=2&taxon_id=200007517 [last accessed on Dec 03, 2024].

Fulton, Michelle, and Scott A. Hodges. 1999. "Floral isolation between *Aquilegia formosa* and *Aquilegia pubescens*." *Proceedings of the Royal Society of London. Series B: Biological Sciences* 266 (1435):2247-2252.

Grant, Karen A., and Verne Grant. 1968. *Hummingbirds and their flowers*. New York and London: Columbia University Press.

Grant, Verne. 1952. "Isolation and hybridization between *Aquilegia formosa* and *A. pubescens*." *Aliso: A Journal of Systematic and Floristic Botany* 2 (4):341-360.

Grant, Verne. 1992. "Floral isolation between ornithophilous and sphingophilous species of *Ipomopsis* and *Aquilegia*." *Proceedings of the National Academy of Sciences* 89 (24):11828-11831.

Grant, Verne. 1993. "Origin of floral isolation between ornithophilous and sphingophilous plant species." *Proceedings of the National Academy of Sciences* 90 (16):7729-7733.

Grant, Verne, and Ethan J. Temeles. 1992. "Foraging ability of rufous hummingbirds on hummingbird flowers and hawkmoth flowers." *Proceedings of the National Academy of Sciences* 89 (20):9400-9404.

Han, Meng, Qing-Qing Zhu, Li Sun, Chen-Yu Niu, Ying Li, Ning Wang, Xiao-Hui Zhang, and Yi Ren. 2022. "Petal ontogeny, structure, and pollination system of four *Aquilegia* species in Midwest China." *Flora* 286:151987.

Hattori, Mitsuru, Yusuke Nagano, and Takao Itino. 2014. "Geographic flower trait variation of *Aquilegia buergeriana* Sieb. et Zucc. var. *buergeriana* on Mt. Norikura and the Utsukushigahara Highland, Japan." *American Journal of Plant Sciences* 2014.

Hodges, Scott A., Michelle Fulton, Ji Y. Yang, and Justen B. Whittall. 2004. "Verne Grant and evolutionary studies of *Aquilegia*." *New Phytologist* 161 (1):113-120. doi: https://doi.org/10.1046/j.1469-8137.2003.00950.x.

Huang, Lei, Fang-Dong Geng, Jing-Jing Fan, Wei Zhai, Cheng Xue, Xiao-Hui Zhang, Yi Ren, and Ju-Qing Kang. 2022. "Evidence for two types of *Aquilegia ecalcarata* and its implications for adaptation to new environments." *Plant Diversity* 44 (2):153-162.

Jäger, Eckehart J., Friedrich Ebel, Peter Hanelt, and Gerd K Müller, eds. 2016. *Rothmaler. Exkursionsflora von Deutschland. Band 5. Krautige Zier- und Nutzpflanzen*. Berlin, Heidelberg: Springer-Verlag.

Kramer, Elena M. 2009. "*Aquilegia*: a new model for plant development, ecology, and evolution." *Annual Review of Plant Biology* 60:261-277.

Kramer, Elena M., and Scott A. Hodges. 2010. "*Aquilegia* as a model system for the evolution and ecology of petals." *Philosophical Transactions of the Royal Society B: Biological Sciences* 365 (1539):477-490.

Kurzenko, Nick. 2012. "*Aquilegia oxysepala* Photo ID: 0000 0000 0911 1884 (2011-09-19)." Regents of the University of California. https://calphotos.berkeley.edu/ [last accessed Oct 13, 2020].

Lauber, Konrad, Gerhart Wagner, and Andreas Gygax. 2018. *Flora Helvetica. Illustrierte Flora der Schweiz*. 6th ed. Bern: Haupt Verlag.

Lavergne, Sebastien, Max Debussche, and John D. Thompson. 2005. "Limitations on reproductive success in endemic *Aquilegia viscosa* (Ranunculaceae) relative to its widespread congener *Aquilegia vulgaris*: the interplay of herbivory and pollination." *Oecologia* 142:212-220.

Ledbetter, Trevor, Sarah Richman, Rebecca Irwin, and Judith Bronstein. 2022. "What are the plant reproductive consequences of losing a nectar robber?" *Journal of Pollination Ecology* 31:97109.

Leimberger, Kara G., Bo Dalsgaard, Joseph A. Tobias, Christopher Wolf, and Matthew G. Betts. 2022. "The evolution, ecology, and conservation of hummingbirds and their interactions with flowering plants." *Biological Reviews* 97 (3):923-959.

LoPresti, E. F., J. Goidell, J. M. Mola, M. L. Page, C. D. Specht, C. Stuligross, M. G. Weber, N. M. Williams, and R. Karban. 2020. "A lever action hypothesis for pendulous hummingbird flowers: experimental evidence from a columbine." *Annals of Botany* 125 (1):59-65.

Luo, Yan, Andrey S. Erst, Chen-Xuan Yang, Jian-Ping Deng, and Lu Li. 2018. "*Aquilegia yangii* (Ranunculaceae), a new species from western China." *Phytotaxa* 348 (4):289-296.

Macior, Lazarus Walter. 1966. "Foraging behavior of *Bombus* (Hymenoptera: Apidae) in relation to *Aquilegia* pollination." *American Journal of Botany* 53 (3):302-309.

Martinell, M. Carmen. 2008. "Planta del Mes núm. 2: Corniol dels Ports." *Portal de Biologia de la Conservacío de plantes. Laboratori de Botànica, Facultat de Farmàcia. Universitat de Barcelona*.

Martinell, M. Carmen, Jordi López-Pujol, Cèsar Blanché, Julián Molero, and Llorenç Sàez. 2011a. "Conservation assessment of *Aquilegia paui* (Ranunculaceae): a case study of an extremely narrow endemic." *Oryx* 45 (2):187-190.

Martinell, M. Carmen, Ana Rovira, Cèsar Blanché, and Maria Bosch. 2011b. "Shift towards autogamy in the extremely narrow endemic *Aquilegia paui* and comparison with its widespread close relative *A. vulgaris* (Ranunculaceae)." *Plant Systematics and Evolution* 295:73-82.

Mathew, Brian, and Mike Sinnott. 2003. "Plate 471. *Aquilegia fragrans*." *Curtis's Botanical Magazine* 20 (3):147-151.

Mayer, Alfred. 2015. *Flora Escursionistica dell'Italia. Prima Parte. Italia Centrale*: Independent Scientific Editions.

Medrano, M., M. C. Castellanos, and C. M. Herrera. 2006. "Comparative floral and vegetative differentiation between two European *Aquilegia* taxa along a narrow contact zone." *Plant Systematics and Evolution* 262:209-224.

Miller, Russell B. 1978. "The pollination ecology of *Aquilegia elegantula* and *A. caerulea* (Ranunculaceae) in Colorado." *American Journal of Botany* 65 (4):406-414.

Miller, Russell B. 1985. "Hawkmoth pollination of *Aquilegia chrysantha* (Ranunculaceae) in southern Arizona." *The Southwestern Naturalist* 30 (1):69-76.

Miller, Russell B., and Cynthia L. Willard. 1983. "The pollination ecology of *Aquilegia micrantha* (Ranunculaceae) in Colorado." *The Southwestern Naturalist* 28 (2):157-164.

Misaki, Ando, Tomoyuki Itagaki, Yutaka Matsubara, and Satoki Sakai. 2018. "Intraflower variation in nectar secretion: Secretion patterns and pollinator behavior in male-and female-phase flowers." *American Journal of Botany* 105 (5):842-850.

Müller, Hermann. 1881. *Alpenblumen, ihre Befruchtung durch Insekten und ihre Anpassungen an dieselben*: W. Engelmann.

Munz, Philip A. 1946. "*Aquilegia*. The Cultivated and Wild Columbines." *Gentes Herbarum* 7:1-150.

Nold, Robert. 2003. *Columbines. Aquilegia, Paraquilegia, and Semiaquilegia*. Portland, Cambridge: Timber Press.

Prazmo, W. 1965. "Cytogenetic studies on the genus *Aquilegia*." *Acta Societatis Botanicorum Poloniae* 34 (3):403-437.

Rodriguez-Flores, Claudia I., Juan Francisco Ornelas, Susan Wethington, and María del Coro Arizmendi. 2019. "Are hummingbirds generalists or specialists? Using network analysis to explore the mechanisms influencing their interaction with nectar resources." *PloS one* 14 (2):e0211855.

Sapir, Nir, and Robert Dudley. 2013. "Implications of floral orientation for flight kinematics and metabolic expenditure of hover-feeding hummingbirds." *Functional Ecology* 27 (1):227-235.

Sikora, Robert. 2015. "*Aquilegia eximia* Photo ID: 0000 0000 0815 0005." Regents of the University of California. https://calphotos.berkeley.edu/ [last accessed Dec 03, 2024].

Spellenberg, Richard. 2020. "*Aquilegia elegantula* Photo ID: 0000 0000 0520 1614." Regents of the University of California. https://calphotos.berkeley.edu/ [last accessed Dec 03, 2024].

Tang, L. L., Q. Yu, J. F. Sun, and S. Q. Huang. 2007. "Floral traits and isolation of three sympatric *Aquilegia* species in the Qinling Mountains, China." *Plant Systematics and Evolution* 267:121-128.

Tela botanica. 2011. "Projet de numérisation de la flore de L'Abbé Coste. *Aquilegia pyrenaica* DC.". https://www.tela-botanica.org/bdtfx-nn-74964-illustrations [last accessed Dec 03, 2024].

Thorsted, Steven. 2001. "*Aquilegia pubescens* Photo ID: 0000 0000 1001 0128." Regents of the University of California. https://calphotos.berkeley.edu/ [last accessed Dec 03, 2024].

Tidestrom, Ivar. 1910. "Species of *Aquilegia* Growing in Utah and in Adjacent Portions of Colorado, Idaho and Arizona." *The American Midland Naturalist* 1 (7):165-171.

Toji, Tsubasa, Shun K. Hirota, Natsumi Ishimoto, Yoshihisa Suyama, and Takao Itino. 2022. "Intraspecific independent evolution of floral spur length in response to local flower visitor size in Japanese *Aquilegia* in different mountain regions." *Ecology and Evolution* 12 (3):e8668.

Trnkoczy, Amadej. 2004. "*Aquilegia vulgaris* Photo ID: 0000 0000 0304 0694." Regents of the University of California. https://calphotos.berkeley.edu/ [last accessed Dec 03, 2024].

Trnkoczy, Amadej. 2020. "*Aquilegia atrata* Photo ID: 0000 0000 0220 0007." Regents of the University of California. https://calphotos.berkeley.edu/ [last accessed Dec 03, 2024].

von Balthazar, Maria, Margarita Lachmayer, Anna-Sophie Hawranek, Constantin Kopper, Jürg Schönenberger, and Marion Chartier. 2025. "Pollination and Reproductive Systems in Columbines (*Aquilegia*, Ranunculaceae): Review and Insights." *International Journal of Plant Sciences* 186 (1):38-52. doi: 10.1086/733070.

von Kirchner, Oskar. 1911. *Blumen und Insekten: ihre Anpassungen aneinander und ihre gegenseitige Abhängigkeit*. Leipzig, Berlin: Teubner.

Whittall, Justen B., and Scott A. Hodges. 2007. "Pollinator shifts drive increasingly long nectar spurs in columbine flowers." *Nature* 447 (7145):706-709.

Xue, Cheng, Fang-Dong Geng, Xiao-Yan Zhang, Xiao-Peng Chang, Ju-Qing Kang, Lei Huang, Jian-Qiang Zhang, and Yi Ren. 2019. "Morphological variation pattern of *Aquilegia ecalcarata* and its relatives." *Journal of Systematics and Evolution* 58 (3):221-233. doi: https://doi.org/10.1111/jse.12494.

Zemenick, Ash T., Jay A. Rosenheim, and Rachel L. Vannette. 2018. "Legitimate visitors and nectar robbers of *Aquilegia formosa* have different effects on nectar bacterial communities." *Ecosphere* 9 (10):e02459.
